# A large‐scale single‐cell transcriptomic atlas indicates the immune panorama of influenza A infection

**DOI:** 10.1002/imt2.70121

**Published:** 2026-03-25

**Authors:** Yi Wang, Shuzi Liu, Laurence Don Wai Luu, Yongzhi Zhai, Chenliang Zhu, Zhaomin Feng, Yao Tan, Linglong Wan, Jie Wang, Juan Zhou, Jing Wang, Lixin Xie, Quanyi Wang, Fei Xie

**Affiliations:** ^1^ Experimental Research Center & Molecular Diagnostic Center, Capital Center for Children's Health Capital Medical University, Capital Institute of Pediatrics Beijing China; ^2^ Beijing Research Center for Respiratory Infectious Diseases Beijing China; ^3^ Department of Respiratory and Critical Care Medicine, The Eight Medical Center Chinese PLA General Hospital Beijing China; ^4^ School of Biotechnology and Biomolecular Sciences, University of New South Wales Sydney Australia; ^5^ Department of Emergency, The First Medical Center Chinese PLA General Hospital, Medical School of Chinese PLA Beijing China; ^6^ Department of Clinical Laboratory, Institute of Translational Medicine Renmin Hospital of Wuhan University Wuhan China; ^7^ Beijing Key Laboratory of Surveillance, Early Warning and Pathogen Research on Emerging Infectious Diseases, Beijing Research Center for Respiratory Infectious Diseases, Beijing Center for Disease Prevention and Control Beijing China; ^8^ College of Pulmonary and Critical Care Medicine, Chinese PLA General Hospital Beijing China; ^9^ Department of Respiratory and Critical Care Medicine Beijing Chaoyang Hospital Affiliated to Capital Medical University Beijing China

**Keywords:** cytokine storm, influenza A virus, peripheral immune response, single‐cell transcriptomic atlas, T cell exhaustion

## Abstract

Influenza A virus (IAV) infection has a wide clinical spectrum, from mild illness to life‐threatening pneumonia, yet the underlying immune determinants of disease remain poorly defined. Here, we generated a large‐scale single‐cell transcriptomic atlas from peripheral blood, profiling more than 612,010 cells from 97 individuals, including healthy controls, and patients with mild, severe, or convalescent IAV infection. Our findings uncovered a core immune dichotomy that determines clinical severity: a protective, monocyte‐centric antiviral state in mild disease versus a pathological, neutrophil‐ and myeloid‐derived suppressor cell (MDSC)‐driven hyperinflammatory state in severe infection. Severe disease was marked by a peripheral hyperinflammatory state, driven by specific monocyte and neutrophil subsets via the *S100A8*/*9*/12–*TLR4*/*RAGE* signaling axis, and was coupled with the expansion of granulocytic MDSCs that likely contribute to T cell paralysis. In contrast, mild disease was associated with a protective, monocyte‐centric response characterized by robust antiviral interferon signaling and enhanced antigen presentation. This functional divergence extends to the adaptive immune system, where mild disease was associated with CD8^+^ T cells displaying a balance of high cytotoxicity and regulated exhaustion. In severe illness, however, T cells become profoundly dysfunctional, exhibiting signatures of metabolic stress and apoptosis alongside the emergence of pathogenic, pro‐inflammatory regulatory T cells. Together, our atlas provides a high‐resolution immunological blueprint of human IAV infection, delineates the cellular states and pathways that govern clinical trajectories and offers a critical resource for developing host‐directed therapies.

## Highlights

- scRNA‐seq analysis indicates a core immune dichotomy in influenza infection, distinguishing a protective monocyte‐centric trajectory in mild disease from a pathological granulocyte‐driven state in severe cases.
- Severe infection is characterized by a hyperinflammatory storm mediated by the *S100A8/9/12*–*TLR4/RAGE* signaling axis, coupled with immune paralysis driven by the expansion of myeloid‐derived suppressor cells.
- Severe disease is marked by profound T cell dysfunction linked to metabolic collapse and mitochondrial failure, alongside the emergence of pathogenic, pro‐inflammatory regulatory T cells.

## Graphical abstract

The study presents a large‐scale single‐cell transcriptomic atlas profiling over 612,010 peripheral immune cells from 97 individuals to decode the heterogeneity of influenza infection. These findings indicate a fundamental immune dichotomy determining clinical trajectories: a protective, monocyte‐centric antiviral state in mild disease versus a pathological, neutrophil‐ and myeloid‐derived suppressor cell (MDSC)‐driven hyperinflammatory state in severe infection. Mechanistically, severe disease is orchestrated by the *S100A8/9/12*–*TLR4/RAGE* axis and MDSC expansion, driving a “double hit” of cytokine storm and immune paralysis. This dysregulated environment precipitates metabolic collapse in effector T cells and the pathogenic conversion of regulatory T cells. Collectively, these results define the cellular checkpoints governing influenza outcomes and highlight host‐directed therapeutic targets.

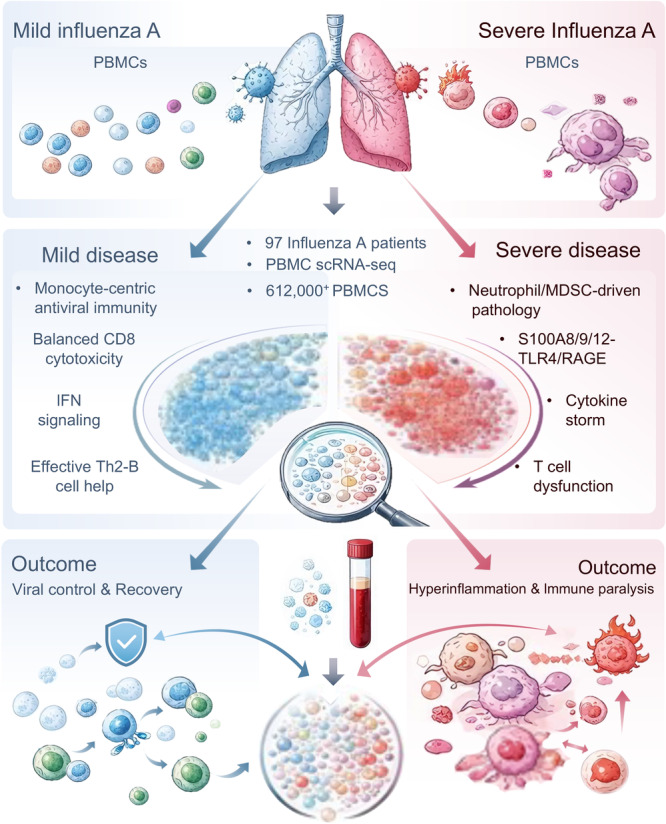

## INTRODUCTION

Influenza viruses are the primary etiological agents of acute respiratory infections with a substantial global health burden [1, 2]. These enveloped RNA viruses are antigenically classified into four types (A, B, C, and D) based on their conserved nucleoprotein and matrix proteins [3, 4]. Of these, influenza A viruses (IAVs) pose the greatest public health threat due to their extensive genetic diversity, broad host range, and capacity to cause both recurrent seasonal epidemics and devastating pandemics [5, 6]. While influenza B viruses (IBVs) also contribute to seasonal disease, their clinical impact is typically less severe [7]. Influenza C and D viruses are also associated with only mild or subclinical infections and are not considered major public health concerns [8].

IAV infections cause substantial morbidity and mortality through seasonal epidemics, driven by efficient airborne transmission patterns, and pose a persistent pandemic threat owing to their rapid viral adaptation and continual antigenic evolution [1, 9, 10]. Although essential, existing countermeasures, including primarily vaccination and antiviral drugs, contain limitations. Seasonal vaccine efficacy is frequently compromised by antigenic mismatch between vaccine and circulating strains, while the therapeutic utility of antiviral drugs is threatened by the emergence of resistant variants [11]. Therefore, a high‐resolution understanding of the host immune response to IAV is urgently needed to inform the development of novel host‐directed therapies and broadly protective universal vaccines.

The canonical immune response to IAV is mediated by a coordinated interplay between the innate and adaptive immune systems [12]. Upon viral entry, innate sentinel cells of the respiratory tract (e.g., epithelial and resident myeloid cells) recognize IAV and initiate a rapid antiviral program marked by the production of type I and III interferons (IFNs) and pro‐inflammatory cytokines [12]. This initial cytokine and chemokine milieu recruits innate effector cells (e.g., neutrophils, monocytes, and natural killer (NK) cells) to contain viral dissemination. Subsequently, antigen‐presenting cells, primarily dendritic cells, migrate to draining lymph nodes to orchestrate the adaptive immune response, which culminates in the expansion of cytotoxic T lymphocytes (CTLs) to clear infected cells and the differentiation of B cells into antibody‐secreting plasma cells [13]. The tightly regulated coordination of these cellular and molecular events is essential for effective viral clearance and the generation of long‐lived immunological memory [13].

Currently, the canonical model does not account for the wide clinical spectrum of IAV infection, which ranges from asymptomatic cases to severe pneumonia and acute respiratory distress syndrome (ARDS) [14]. The specific immune cell subsets, dynamic interactions, and molecular networks that differentiate protective from pathological host responses remain largely unresolved. Conventional methodologies (e.g., bulk transcriptomics and flow cytometry) lack the resolution to decipher this cellular and transcriptional complexity. Bulk analyses average gene expression across heterogeneous cell populations, masking the contributions of rare but functionally critical subsets [15], whereas flow cytometry is limited to interrogating a pre‐defined panel of proteins, precluding comprehensive transcriptional profiling.

Single‐cell RNA sequencing (scRNA‐seq) is a transformative technology that dissects cellular heterogeneity and transcriptional states with unprecedented resolution [16, 17]. By quantifying thousands of transcripts in individual cells, this technology is well‐suited to studying the heterogeneity of immune responses and interactions between immune cells, other host cells and pathogens [18]. scRNA‐seq facilitates the unbiased identification of cell populations, mapping of differentiation trajectories, and inference of intercellular communication networks [19], and has been valuable in defining the immune landscape of other major infectious diseases, including COVID‐19 [17], bacterial pneumonia [20, 21], and tuberculosis [22]. However, a comprehensive scRNA‐seq atlas of the immune response during human IAV infection has been lacking. Existing studies have been limited to small sample sizes or focused on isolated cell lineages, failing to capture the systemic intercellular communication networks that drive disease progression [23, 24]. Consequently, a holistic view of the immune landscape remains elusive.

Here, to bridge this knowledge gap, we present the first large‐scale single‐cell transcriptomic atlas of peripheral immune response to human IAV infection. Profiling peripheral blood mononuclear cells (PBMCs) from a cohort of IAV‐infected patients and healthy donors, we delineate the cellular composition and transcriptional programs of the immune response, and provide a foundational immune blueprint for human IAV infection. Analysis of this atlas indicated key immune signatures and intercellular communication networks associated with disease severity. Specifically, the core objective of this study was to analyze the dynamics of peripheral immune cell subsets and key regulatory pathways in mild and severe IAV infections, and to provide an experimental basis for targeted immunotherapy. This atlas provides a high‐resolution landscape of the human immune response to IAV and identifies candidate therapeutic targets and biomarkers for patient stratification.

## RESULTS

### Single‐cell transcriptomics defines the immune panorama during IAV infection

**FIGURE 1:**
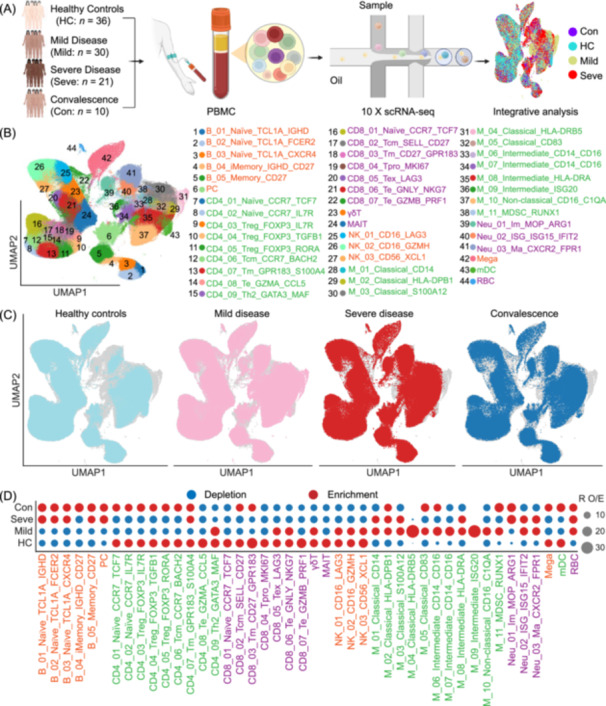
A single‐cell atlas of the peripheral immune response to influenza A virus infection. (A) Schematic overview of the study design. Peripheral blood was collected from 97 individuals across four groups: healthy controls (HC, *n* = 36), patients with mild IAV infection (Mild, *n* = 30), patients with severe IAV infection (Seve, *n* = 21), and convalescent patients (Con, *n* = 10). Peripheral blood mononuclear cells (PBMCs) were isolated and profiled using 10 × Genomics single‐cell RNA sequencing (scRNA‐seq), followed by integrative computational analysis. (B) Uniform Manifold Approximation and Projection (UMAP) visualization of 612,010 high‐quality cells from all 97 donors, colored and numbered to indicate 44 distinct immune cell subsets. The legend provides the detailed annotation for each cluster. (C) UMAP projections of all cells, split and colored by clinical group: Healthy controls (light blue), Mild disease (pink), Severe disease (red), and Convalescence (dark blue). (D) Dot plot illustrating the compositional changes of the 44 cell subsets across the four clinical groups (HC: *n* = 36; Mild: *n* = 30; Severe: *n* = 21; Convalescent, *n* = 10). The ratio of observed to expected (*R*_O/E_) cell counts was calculated as observed cell count/expected cell count, where the expected count assumes a uniform distribution based on total library size. The color scale indicates depletion (blue) or enrichment (red) of a given cell population relative to the expected frequency. The size of the dot corresponds to the magnitude of the *R*_O/E_ value. Cell subsets are grouped by major lineage on the *x*‐axis.

To dissect the peripheral immune response during IAV infection, we performed droplet‐based single‐cell RNA sequencing (scRNA‐seq; 10×Genomics) on blood samples from a cohort of 97 individuals. This cohort included healthy controls (HC; *n* = 36), patients with mild (*n* = 30) or severe (*n* = 21) disease, and convalescent patients recovering from severe disease (*n* = 10) (Figure 1A). Detailed clinical characteristics and laboratory data are provided in Table S1 and Figure S1. Patients with severe disease were significantly older than those with mild disease (Figure S1E), consistent with advanced age as a risk factor for disease severity [25]. We initially profiled 761,305 cells from the 97 samples (Figure S1A–D). Following computational doublet removal and rigorous data filtration (see Methods), we obtained a final dataset of 612,010 high‐quality cells, with an average of 6314 unique molecular identifiers (UMIs) and 1958 detected genes per cell (Figure S1A–D). After normalization for technical confounders like read depth and mitochondrial gene content, high‐quality cells were integrated into a unified, batch‐corrected dataset for principal component analysis (see Methods). This final dataset was composed of 191,925 cells (31.36%) from healthy controls, 212,834 (34.78%) from mild patients, 136,969 (22.38%) from severe patients, and 70,282 (11.48%) from convalescent patients.

Unsupervised graph‐based clustering and Uniform Manifold Approximation and Projection (UMAP) visualization of the integrated dataset identified 12 principal immune cell lineages based on canonical gene marker expression (Figure S2A,B and Table S2). These included B cells, plasma cells (PC), CD4^+^ T cells, CD8^+^ T cells, mucosal‐associated invariant T (MAIT) cells, γδ T cells, natural killer (NK) cells, neutrophils, monocytes, myeloid dendritic cells (mDCs), megakaryocytes (Mega), and red blood cells (RBCs). The UMAP embedding displayed T and NK lymphoid populations separated from B cells, while neutrophils and monocytes clustered distinctly from the lymphoid compartment (Figure 1B, Figure S2A). When UMAP projections were colored by clinical status, cells segregated by disease severity, with severe patients exhibiting a distinct distribution compared to HCs, mild, and convalescent groups, highlighting substantial transcriptomic reprogramming driven by IAV infection (Figure 1C).

To achieve a more granular definition of cellular states and transcriptional alterations elicited by IAV infection, we performed iterative sub‐clustering on key major cell populations. This analysis, guided by canonical markers and cluster‐defining genes, resolved the major lineages into 44 distinct cell subsets, including 9 CD4^+^ T cell, 7 CD8^+^T cell, 3 NK cell, 11 monocyte, 5 B cell, and 3 neutrophil subsets (Figure 1B and Table S2). We annotated these subsets based on representative marker genes, which were incorporated into their nomenclature (e.g., CD4_01_Naïve_CCR7_TCF7). Notably, our dataset captured neutrophils and RBCs, populations typically depleted during standard PBMC isolation. The presence of these cells in IAV patients is consistent with findings in other severe infectious diseases and likely reflects the co‐purification of density‐altered immature or activated neutrophils and progenitor cells mobilized from the bone marrow during systemic inflammation [26]. The ability to capture these biologically relevant populations supports the robustness of our cell isolation and data processing pipeline. Thus, our study provides a high‐resolution cellular atlas of the peripheral immune response to IAV, serving as a valuable resource for dissecting cell‐type‐specific dynamics in health and disease.

To delineate the immunological signatures of disease severity, we quantified the relative abundance of each cell population across the patient cohort. This analysis indicated significant shifts in cellular composition that correlated with disease status (Figure 1D, Figure S2C,D). Influenza patients exhibited a marked reduction in T and NK cell frequencies compared to healthy controls, a lymphopenia that was most pronounced in severe cases. This T and NK cell depletion in severe patients did not fully resolve during convalescence, suggesting it is a key immunological feature of severe illness. In contrast to the depletion of T and NK cells, the frequencies of B cells and plasma cells (PCs) were significantly elevated in patients with severe disease. Most myeloid populations (excluding dendritic cells) also expanded during IAV infection; however, the increase in monocyte frequency was greater in mild compared to severe cases. This pattern contrasts with severe COVID‐19, where monocyte populations are markedly elevated [15, 17], suggesting distinct myeloid dynamics in IAV infection. Similar to the lymphopenia observed, these elevated myeloid fractions persisted into the convalescent phase. Importantly, these alterations in immune cell proportions were consistent with clinical complete blood count data from the same individuals, validating the robustness of our single‐cell approach (Figures S1H–J, S2).

A more granular analysis of compositional shifts, using a ratio of observed‐to‐expected (*R*_O/E_) cell counts, indicated subpopulation‐specific dynamics (Figure 1D). Severe disease was characterized by a profound depletion across most T and NK cell subsets, accompanied by a marked enrichment of B cell subsets. Mild disease, in contrast, was primarily characterized by an enrichment of specific monocyte subsets, particularly activated classical monocytes (e.g., M_04_Classical_HLA‐DR) and interferon‐stimulated intermediate monocytes (e.g., M_09_Intermediate_ISG20). This distinct monocyte signature in mild disease suggests that robust monocyte activation and interferon signaling may be associated with effective viral control and the prevention of severe disease. Together, these results suggest that IAV infection is characterized by discrete, severity‐dependent immunological profiles in the peripheral blood.

### Monocytes and neutrophils orchestrate the peripheral inflammatory storm in severe patients

**FIGURE 2:**
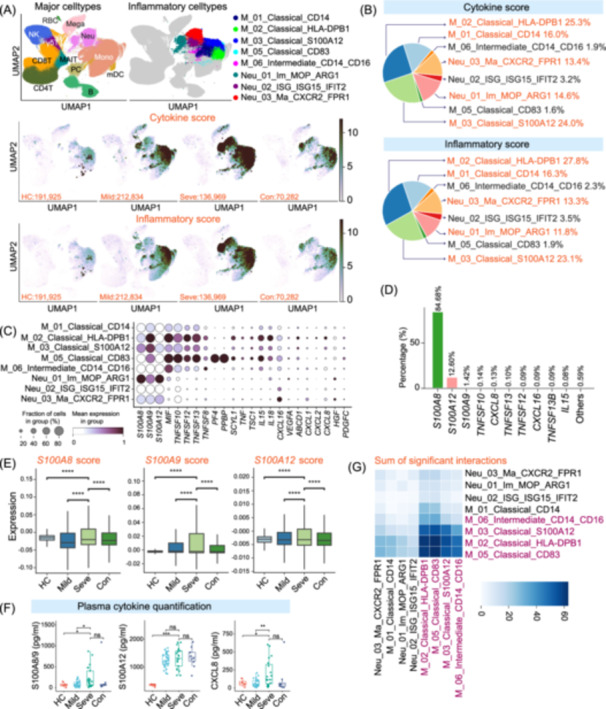
Hyperinflammatory monocytes and neutrophils drive the cytokine storm in severe IAV infection. (A) Uniform Manifold Approximation and Projection (UMAP) visualizations showing the eight identified hyperinflammatory myeloid cell subsets (top right), distinct from other major immune lineages (top left). Bottom panels are UMAP feature plots illustrating the per‐cell Cytokine Score (middle) and Inflammatory Score (bottom) across the four clinical groups, with cell counts indicated for each group. The color scale represents the score intensity. (B) Pie charts showing the relative contribution of each of the eight hyperinflammatory myeloid subsets to the total Cytokine Score (top) and Inflammatory Score (bottom) calculated from all cells in severe patients. (C) Dot plot showing the expression of key pro‐inflammatory genes within the eight hyperinflammatory myeloid subsets. The size of the dot represents the percentage of cells expressing the gene, and the color intensity indicates the average expression level. (D) Bar plot showing the percentage contribution of the top ten pro‐inflammatory cytokines to the total cytokine score in severe patients, highlighting the dominant role of S100 proteins. (E) Box plots comparing the gene module scores for *S100A8*, *S100A9*, and *S100A12* across the four clinical groups (HC, Mild, Seve, Con). (F) Box plots showing plasma concentrations (pg/mL) of the *S100A8/9* complex, *S100A12*, and *CXCL8* as measured by ELISA in individuals from the four clinical groups. (G) Heatmap visualizing the overall communication strength among the eight hyperinflammatory myeloid subsets in severe patients. The color scale indicates the sum of significant ligand‐receptor interaction weights for each cell–cell pair. For all box plots, n represents the number of individuals (HC = 36, Mild = 30, Severe = 21, Convalescent = 10). The center line represents the median, the box limits represent the upper and lower quartiles, and the whiskers extend to 1.5 times the interquartile range. Asterisks denote statistical significance (*p < 0.05, **p < 0.01, ***p < 0.001, ****p < 0.0001; ns, not significant) as determined by Kruskal–Wallis test with Bonferroni correction. IAV, influenza A virus.

Severe patients exhibited markedly elevated levels of C‐reactive protein, indicative of a pronounced systemic inflammatory state (Figure S1G). To delineate the cellular sources driving the inflammation, we calculated the cytokine and inflammatory scores for each immune cell based on collected sets of pro‐inflammatory and inflammatory response genes, respectively (Table S3). Consistent with the clinical findings, both scores were significantly elevated in PBMCs from patients with severe disease compared to those with mild disease, convalescent individuals, and healthy controls (Figure 2A, Figure S3A). This transcriptional signature highlights a potential peripheral inflammatory storm in severe patients, providing a molecular correlate for disease severity.

scRNA‐seq analysis identified 13 myeloid cell subtypes (10 monocyte and 3 neutrophil) characterized by significantly elevated cytokine and inflammatory scores, suggesting their role as potential sources of the inflammatory storm (Figure S3B). Among these, we further distinguished eight subtypes: five monocyte (one classical, M_01_Classical_CD14; three activated classical, M_02_Classical_HLA‐DPB1, M_03_Classical_S100A12, M_05_Classical_CD83; and one intermediate, M_06_Intermediate_CD14_CD16) and three neutrophil (one immature, Neu_01_Im_MOP_ARG1; one ISG‐high, Neu_02_ISG_ISG15_IFIT2; and one mature, Neu_03_Ma_CXCR2_FPR1), which exhibited markedly increased cytokine and inflammatory scores in severe patients compared with other groups (Figure S3C), implicating them as the principal orchestrators of the peripheral cytokine storm. Intriguingly, their relative abundance did not significantly increase in severe cases (Figure S3D), indicating that a heightened per‐cell inflammatory activity, rather than an expansion in cell number, was associated with the intensified peripheral inflammatory response.

Further analysis indicated that five of the hyperinflammatory subtypes (M_01_Classical_CD14, M_02_Classical_HLA‐DPB1, M_03_Classical_S100A12, Neu_01_Im_MOP_ARG1, and Neu_03_Ma_CXCR2_FPR1) accounted for over 92% of the total inflammatory and cytokine scores in severe patients (Figure 2B), confirming their dominant role in driving peripheral inflammation. This finding is consistent with previous studies implicating monocytes and neutrophils as key drivers of inflammation in infectious diseases such as COVID‐19 [15, 17]. Gene expression analysis of these subtypes indicated distinct pro‐inflammatory profiles, characterized by high expression of key genes such as *S100A8/9/12*, *TNF*, and *CXCL1/2/8* (Figure 2C), indicating that diverse molecular mechanisms underpin the peripheral storm.

We identified 10 key pro‐inflammatory cytokines (*S100A8/9/12, TNFSF10/12/13/13B, CXCL8/16*, and *IL15*) that collectively accounted for over 99% of the total cytokine score in severe patients (Figure 2D) which were predominantly expressed by the aforementioned monocyte and neutrophil subtypes (Figure S4A). Among these, *S100A8/9/12* were the core contributors, responsible for approximately 98.7% of the total cytokine score (Figure 2D), and their expression was markedly higher in severe patients compared with other groups (Figure 2E). Other cytokines (e.g., *CXCL8*) may act synergistically with *S100* proteins to exacerbate inflammation. To provide orthogonal validation of these transcriptomic signatures at the protein level, plasma analysis indicated significantly elevated concentrations of the *S100A8/9* complex and *S100A12* in severe patients (Figure 2F), confirming that the upregulation of these alarmins translates into systemic inflammation. Collectively, these findings highlight hyperinflammatory monocytes and neutrophils and their products, particularly *S100A8/9/12*, as potential therapeutic targets.

Previous mechanistic studies have firmly established that *S100A8/9/12* proteins exert pro‐inflammatory effects via *TLR4* and *RAGE* signaling [27, 28], triggering NF‐κB activation and inflammatory cytokine secretion [29]. Building on this established biology, we found that *TLR4*, *AGER* (*RAGE*), and *MYD88* were highly expressed in hyperinflammatory subsets from severe patients (Figure S4B). Statistical analysis confirmed that the expression scores of these signaling components were significantly upregulated in severe patients compared to mild cases and healthy controls (Figure S4C). This co‐expression pattern suggests a potential S100‐driven autocrine/paracrine feedback loop that amplifies the inflammatory cascade. Thus, targeting the *S100*–*TLR4*/*RAGE* signaling axis represents another promising therapeutic strategy for mitigating this hyperinflammatory state.

To further elucidate the cell‐to‐cell communication driving the hyperinflammatory state in severe patients, we analyzed ligand–receptor (L–R) interaction patterns among the eight identified hyperinflammatory subtypes. Our analysis indicated a dense network of both intra‐ and inter‐subtype interactions (Figure 2G, Figure S5). Among these, four monocyte subtypes, three activated classical (M_02_Classical_HLA‐DPB1, M_03_Classical_S100A12, M_05_Classical_CD83) and one intermediate (M_06_Intermediate_CD14_CD16), emerged as major communication hubs, displaying the most significant interaction strengths (Figure 2G). They expressed a repertoire of receptors, including *NOTCH1*, *CCR1*, *CXCR1*, *CXCR2*, and *IL15RA*, indicating their capacity to respond to a wide array of signals from their microenvironment (Figure S5). Across the network, interactions were largely mediated by key pro‐inflammatory axes, including chemokine–receptor pairs (e.g., *CXCL8*–*CXCR1*/*2*, *CCL3*–*CCR1*) and members of the TNF superfamily with their cognate receptors (e.g., *TNF*–*TNFRSF1A*/*B*) (Figure S5). These findings offer a molecular blueprint of the complex interplay among hyperinflammatory subtypes that contributes to severe peripheral immunopathology.

Together, our findings identified specific hyperinflammatory monocyte and neutrophil subpopulations as principal drivers of the peripheral cytokine storm in severe patients, primarily via the *S100A8/9/12* axis and complex intercellular communication networks. Interventions targeting the hyperinflammatory subtypes or their key effector pathways hold therapeutic potential for mitigating systemic immunopathology.

### Distinct CD8^+^ T lymphocyte trajectories distinguish disease severity

**FIGURE 3:**
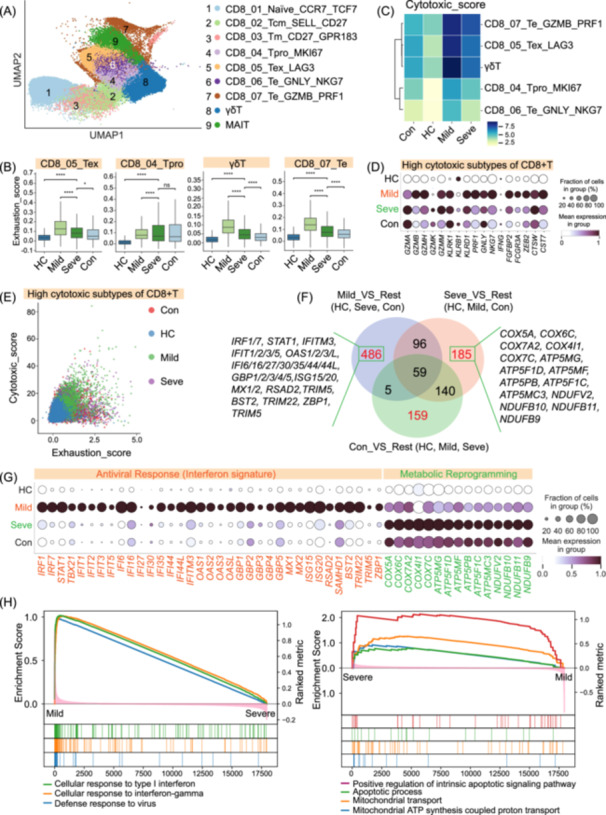
Dysfunctional CD8^+^ T cell states are associated with severe IAV infection. (A) UMAP visualization of 9 distinct CD8^+^ T cell, MAIT, and γδ T cell subsets identified by sub‐clustering. (B) Box plots showing the exhaustion module scores for four key CD8^+^ T cell and γδ T cell subsets across the four clinical groups (HC, Mild, Seve, Con). (C) Heatmap displaying the hierarchical clustering of selected CD8^+^ T cell and γδ T cell subsets based on their average cytotoxic module score in each clinical group. The color scale indicates the score intensity. (D) Dot plot showing the expression of key cytotoxic effector genes in the high‐cytotoxicity CD8^+^ T cell subsets across the four clinical groups. Dot size represents the fraction of cells expressing the gene, and color intensity indicates the average expression level. (E) Scatter plot showing the distribution of individual CD8^+^ T cells based on their cytotoxic (*y*‐axis) and exhaustion (*x*‐axis) module scores. Each dot represents a single cell, colored by its clinical group of origin. (F) Venn diagram illustrating the overlap of differentially expressed genes (DEGs) in CD8^+^ T cells from Mild, Severe, and Convalescent patients compared to the remaining groups (Rest). Key upregulated genes in each condition‐specific set are listed. (G) Dot plot displaying the expression of representative DEGs associated with antiviral interferon signaling (left) and metabolic reprogramming (right) in CD8^+^ T cells across the four clinical groups. (H) Gene Set Enrichment Analysis (GSEA) plots showing enrichment of key Gene Ontology (GO) terms in CD8^+^T cells. Left panels compare Mild versus Severe patients for interferon‐related pathways. Right panels compare Severe versus Mild patients for pathways related to apoptosis and mitochondrial metabolism. For all box plots, *n* represents the number of individuals (HC = 36, Mild = 30, Severe = 21, Convalescent = 10). The center line represents the median, the box limits represent the upper and lower quartiles, and the whiskers extend to 1.5 times the interquartile range. Asterisks denote statistical significance (*p < 0.05, **p < 0.01, ***p < 0.001, ****p < 0.0001; ns, not significant) as determined by Kruskal–Wallis test with Bonferroni correction. IAV, influenza A virus; MAIT, mucosal‐associated invariant T; UMAP, Uniform Manifold Approximation and Projection.

To dissect CD8^+^ T cell heterogeneity and functional states during IAV infection, we performed sub‐clustering and resolved nine distinct populations: naïve (CD8_01_Naïve_CCR7_TCF7), central memory (Tcm, CD8_02_Tcm_SELL_CD27), memory (Tm, CD8_03_Tm_CD27_GPR183), proliferating (Tpro, CD8_04_Tpro_MKI67), exhausted (Tex, CD8_05_Tex_LAG3), two effector (Te, CD8_06_Te_GNLY_NKG7 and CD8_07_Te_GZMB_PRF1), MAIT, and γδ T cells (Figure 3A, Figure S6A, and Table S2). Analysis of established gene signatures confirmed their functional identities [21, 30], indicating distinct patterns of CD8^+^ T cell subtypes (Figure S6B). Naïve cells had high naïve scores, while Te, Tex, Tpro, and γδ T cells exhibited high cytotoxicity scores. The Tex subset showed the highest exhaustion score, with elevated exhaustion also observed in Tpro, γδ T, and one of the Te subsets (CD8_07_Te).

Notably, overall exhaustion scores for CD8⁺ T cell subsets were significantly higher in mild patients than in severe cases (Figure 3B). This implies that CD8⁺ T cells in mild patients might enter a regulated exhaustion state, potentially reflecting effective initial activation followed by controlled immune dampening. CD8⁺ T cell subsets from mild patients exhibited elevated expression of canonical exhaustion markers (*LAG3*, *TIGIT*, *CD244*, and *CD160*) and related transcriptional regulators known to orchestrate and enforce the T cell exhaustion program, including the master regulator *TOX*, as well as *EOMES*, *NR4A2* (*Nurr1*), and the inhibitory phosphatase *PTPN6* (*SHP‐1*) (Figure S7A). These findings suggest that the exhausted phenotype in mild disease likely represents a transcriptionally regulated, non‐terminal exhaustion program, indicative of an adaptive response to persistent antigen stimulation that retains functional potential rather than irreversible dysfunction.

Hierarchical clustering based on a cytotoxic gene module score indicated that multiple CD8⁺ T cell subsets from patients with mild disease exhibited significantly higher cytotoxic activity than those from severe cases, healthy controls, or convalescent individuals (Figure 3C). This enhanced cytotoxic profile in mild cases was driven by key effector molecules, including granzyme B (*GZMB*), granzyme H (*GZMH*), and granulysin (*GNLY*), suggesting a robust and effective antiviral response (Figure 3D). To investigate the relationship between cytotoxicity and exhaustion, we co‐plotted CD8⁺ T cell subsets based on their respective module scores (Figure 3E). Notably, cells from mild cases were concentrated in a high‐cytotoxicity, high‐exhaustion quadrant, consistent with a functionally regulated effector state. In contrast, CD8⁺ T cells from severe patients showed lower cytotoxicity scores and were broadly distributed across exhaustion levels, indicative of a heterogeneous and potentially dysfunctional response. Conversely, cells from HC were predominantly localized to the low‐cytotoxicity, low‐exhaustion region, reflecting immune quiescence. These findings suggest a model wherein a balanced state of exhaustion in mild disease preserves potent cytotoxic function, whereas severe patients are characterized by a decline in cytotoxicity and dysregulated cellular states.

Differential gene expression (DEG) analysis indicated distinct CD8⁺ T cell molecular programs associated with disease severity and resolution (Figure 3F and Tables S4−6). In mild cases, CD8⁺ T cells exhibited a coordinated upregulation of a transcriptional program encompassing interferon‐stimulated genes (ISGs; e.g., *MX1*, *ISG15*, *STAT1*) (Figure 3F,G), potent cytotoxic effector molecules essential for granule‐mediated killing (e.g., granzyme M (*GZMM*), perforin (*PRF1*)) (Figure 3D), and immunoregulatory molecules (e.g., *TIGIT*) (Figure S7A), indicating a balanced and effective antiviral response. In contrast, CD8⁺ T cells from severe cases displayed a divergent signature characterized by heightened expression of MHC class II molecules (e.g., *HLA‐DRA*, *HLA‐DRB5*), markers of persistent activation and dysfunction (e.g., *PVRIG*, *IL2RA*, *CISH*), and genes linked to mitochondrial metabolic stress and pro‐apoptotic signaling. This included key components of the mitochondrial electron transport chain (e.g., *COX5A*, *ATP5MG*) and critical drivers of programmed cell death (e.g., *BAX*, *PYCARD*) (Figure 3F,G). Finally, the transcriptional profile of cells from convalescent individuals was enriched for genes involved in the establishment of T cell memory and differentiation, such as the chemokine receptor *CXCR3* and the transcription factors *FOXP1*, *JUND*, as well as markers of immune quiescence (e.g., *CTLA4*) (Figure S7B), consistent with immunological resolution.

Gene Ontology (GO) enrichment analyses corroborated these divergent cellular programs (Figure S7C). CD8^+^ T cells in mild disease were enriched in pathways such as “type I interferon signaling pathway” and “defense response to virus,” consistent with an effective antiviral response [31]. In contrast, CD8^+^ T cells from severe cases exhibited enrichment for pathways related to mitochondrial bioenergetics (e.g., “mitochondrial ATP synthesis coupled proton transport,” “aerobic electron transport chain”), “apoptotic process” and “cellular senescence,” indicative of profound metabolic stress and cell death. CD8^+^ T cells from convalescent individuals were enriched in pathways related to “DNA replication” and “chromatin remodeling,” suggesting processes integral to cellular recovery and memory pool establishment, and transcriptional resetting (Tables S7–9). These pathway‐level distinctions were further substantiated by Gene Set Enrichment Analysis (GSEA) (Figure 3H). Together, these findings have delineated the critical CD8⁺ T cell transcriptional states that orchestrate either protective immunity or immunopathology during IAV infection and highlight potential targets for therapeutic intervention.

### CD4^+^ T cell heterogeneity defines disease severity

**FIGURE 4:**
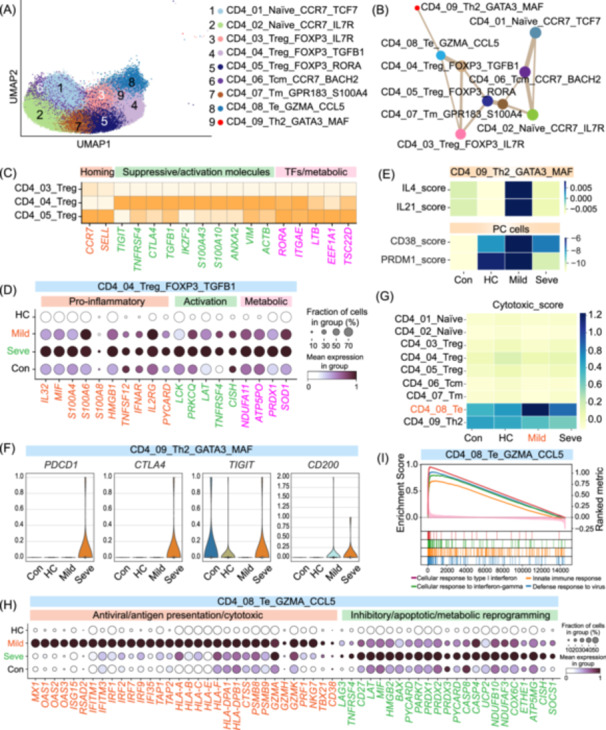
Pathogenic Tregs and dysfunctional effector CD4^+^ T cells characterize severe IAV infection. (A) UMAP visualization of 9 distinct CD4^+^ T cell subsets identified by sub‐clustering. (B) Partition‐based graph abstraction (PAGA) analysis illustrating inferred developmental trajectories among the CD4^+^ T cell subsets. Line thickness corresponds to the strength of the connection. (C) Heatmap displaying the expression of key genes related to homing, suppression/activation, and metabolism/transcription factors (TFs) across the three identified Treg subsets. (D) Dot plot showing the expression of pro‐inflammatory, activation, and metabolic stress‐related genes in the CD4_04_Treg_FOXP3_TGFB1 subset across the four clinical groups. (E) Heatmap displaying module scores for Th2‐associated cytokines (*IL4*, *IL21*) in the CD4_09_Th2_GATA3_MAF subset and plasma cell markers (*CD38*, *PRDM1*) in plasma cells (PCs) across the clinical groups, suggesting a functional link. (F) Violin plots showing the expression of key exhaustion markers (*PDCD1*, *CTLA4*, *TIGIT*, *CD200*) in the CD4_09_Th2_GATA3_MAF subset across the four clinical groups. (G) Heatmap displaying the average cytotoxic module score for each of the nine CD4^+^ T cell subsets across the clinical groups. (H) Dot plot showing the expression of representative genes associated with antiviral/antigen presentation/cytotoxicity and inhibitory/apoptotic/metabolic reprogramming in the CD4_08_Te_GZMA_CCL5 subset across the clinical groups. (I) Gene Set Enrichment Analysis (GSEA) plot showing the enrichment of interferon and antiviral response pathways in CD4_08_Te_GZMA_CCL5 cells from mild compared to severe patients. For dot plots, the size of the dot represents the percentage of cells expressing the gene, and the color intensity indicates the average expression level. IAV, influenza A virus; UMAP, Uniform Manifold Approximation and Projection.

Unbiased transcriptomic clustering of CD4^+^ T cells identified nine distinct subsets (Figure 4A, Figure S8A, and Table S2), including two naïve populations (CD4_01_Naïve_CCR7_TCF7, CD4_02_Naïve_CCR7_IL7R), three regulatory T cell (Treg) clusters (CD4_03_Treg_FOXP3_IL7R, CD4_04_Treg_FOXP3_TGFB1, CD4_05_Treg_FOXP3_RORA), a central memory (Tcm) subset (CD4_06_Tcm_CCR7_BACH2), a memory (Tm) subset (CD4_07_Tm_GPR183_S100A4), an effector (Te) subset (CD4_08_Te_GZMA_CCL5), and a Th2‐like cluster (CD4_09_Th2_GATA3_MAF). To infer their developmental relationships, we employed partition‐based graph abstraction (PAGA), which indicated a principal trajectory extending from naïve and Tcm populations toward a continuum of Treg states (Figure 4B). Notably, this analysis positioned the CD4_04_Treg_FOXP3_TGFB1 subset as an intermediate state linked to both the Te and Th2‐like clusters, suggesting a potential differentiation path toward these effector lineages. Furthermore, this trajectory was mirrored by the functional profiles of the cells: module scoring confirmed high naïveté in naïve cells, strong regulatory potential in Tregs, potent cytotoxicity in Te cells, and prominent exhaustion signatures in the Th2‐like cluster (Figure S8B).

Within the Treg compartment, we identified three populations distinguished by unique molecular signatures (Figure 4C). The CD4_03_Treg_FOXP3_IL7R cluster resembled central Tregs, expressing the lymph node‐homing receptors *CCR7* and *SELL*. In contrast, the CD4_04_Treg_FOXP3_TGFB1 subset represented highly activated effector Tregs (eTregs), characterized by co‐expression of inhibitory (e.g., *TIGIT*, *CTLA4*), co‐stimulatory receptors (e.g., *TNFRSF4*), key regulators (e.g., *TGFB1*, *IKZF2*), and activation‐related genes (e.g., *S100A4*, *VIM*), indicative of strong immunosuppressive potential. The CD4_05_Treg_FOXP3_RORA subset displayed a specialized signature that included the transcription factor *RORA*, tissue‐residency integrin *ITGAE*, and cytoskeletal/metabolic genes (e.g., *ACTB*, *EEF1A1*, *TSC22D3*), suggesting a specialized or terminally differentiated state. To delineate the role of eTregs in disease severity, we focused on the CD4_04_Treg_FOXP3_TGFB1 subset and found that in severe cases, these cells adopted a systemic pathogenic, pro‐inflammatory phenotype (Figure 4D). This state was marked by elevated expression of inflammatory mediators (*IL32*, *MIF*), damage‐associated molecular patterns (DAMPs; *S100A4*/*A6*/*A8*, *HMGB1*), cytokines/receptors (*TNFSF12*, *IFNAR2*, *IL2RG*), and activation/signaling molecules (*LCK*, *PRKCQ*, *LAT*, *TNFRSF4*, *CISH*), potentiating systemic inflammation. Concurrently, these eTregs exhibited profound cellular stress and inflammasome priming, evidenced by upregulation of *PYCARD* (ASC), mitochondrial genes reflecting metabolic strain (e.g., *NDUFA11*, *ATP5PO*, *COX5A*), and oxidative stress responders (*PRDX1*, *SOD1*). GO enrichment analysis further corroborated this hyperactivated and stressed cellular state (Figure S9A). Together, our data suggest that this pathogenic eTreg subset is a key driver of immunopathology in severe cases.

In addition to Tregs, we investigated the roles of effector CD4^+^ T cell subsets, specifically Te and Th2‐like cells, in influenza pathogenesis. Th2‐like cells, defined by high expression of *GATA3* and *MAF* (Figure S8A), were notably more abundant in mild patients compared to severe cases (Figure 1D). In mild disease, these cells upregulated genes associated with humoral immunity, including *IL4* and *IL21* (Figure 4E), which promote B‐cell activation and plasma cell differentiation [32]. Consistent with this function, we observed a coordinated upregulation of these cytokines with the key plasma cell markers *CD38* and *PRDM1* (Figure 4E). GSEA analysis further indicated the activation of “defense response to virus,” “IL‐4 signaling,” and “B‐cell receptor signaling” pathways in Th2‐like cells from mild cases (Figure S9B). In severe illness, the proportion of Th2‐like cells was markedly reduced (Figure 1E) and accompanied by elevated expression of exhaustion markers (*PDCD1*, *CTLA4*, *TIGIT*, and *CD200*) (Figure 4F). This exhausted phenotype corresponded with diminished IL4 and IL21 expression (Figure 4E), potentially impairing antibody production and contributing to viral persistence.

Parallel to the dysregulation of Th2‐like cells, we examined the CD4_08_Te_GZMA_CCL5 (Te) cell compartment, which was characterized by *GZMA* and *CCL5* expression and a broad cytotoxic program that included *PRF1*, *GNLY*, *NKG7*, and *GZMK* (Figure S9C). This cytotoxic potential was pronounced in Te cells from patients with mild disease (Figure 4G), suggesting that a more effective Te response correlates with less severe disease. DEG analysis indicated distinct transcriptional programs, with 287 genes uniquely upregulated in Te cells from mild patients, and 374 genes uniquely upregulated in severe patients (Figure S9D and Tables S10–14). The mild‐specific DEG profile indicated a robustly activated, IFN‐driven state equipped for anti‐viral activity and immune stimulation (Figure 4H). Key genes within this mild‐specific signature included potent antiviral ISGs (*MX1*, *OAS1*/*2*/*3*, *ISG15*, *RSAD2*, *IFITM1*/*3*), molecules for antigen presentation (*TAP1*/*2*, *HLA‐A*/*B*/*C*/*E*/*F*/*DPA1*/*DPB1*, *CTSS*, *PSMB8*/*9*), cytotoxic effectors (*GZMA*/*H*/*K*, *PRF1*, *NKG7*), the Th1‐skewing factor *TBX21*, and activation marker *CD38* (Figure 4H). Notably, broader antiviral responses (e.g., defense, IFN‐I, IFN‐γ) were systemically upregulated across CD4^+^ T cell subsets in mild patients (Figure S9E), likely fostering an environment conducive to effective Te cell function. GO and GSEA further highlighted the enrichment of “type I interferon signaling,” “response to virus,” and “T cell activation” pathways in Te cells from mild patients (Figure 4I, Figure S10A). In contrast, T cells from severe cases upregulated a distinct set of 374 genes indicative of heightened activation coupled with cellular stress and dysfunction. This severe‐specific signature included co‐stimulatory and activation receptors (*TNFRSF4*, *CD27*, *LAT*), inhibitory receptors (*LAG3* and *CTLA4*), stress‐response and pro‐apoptotic genes (*MIF*, *HMGB2*, *BAX*, *PYCARD*, *PARK7*, *PRDX1*/*2*/*3* and *CASP4*/*8*), inflammasome components (*PYCARD*), and genes related to metabolic reprogramming and mitochondrial stress (*UCP2*, *NDUFB10*, *NDUFAF3*, *COX6C*, *ETHE1*, *ATP5MG*) (Figure 4H). Furthermore, negative feedback regulators like *CISH* and *SOCS1* were also upregulated in severe cases. GO analysis of these severe‐specific genes indicated enrichment for pathways including “regulation of apoptotic process,” “mitochondrial ATP synthesis,” “T cell receptor signaling,” and “purine nucleotide metabolism” (Figure S10B). Correspondingly, pathways like “T cell receptor signaling” and “regulation of apoptotic process” were broadly elevated across CD4^+^ T cell subsets in severe disease (Figure S10C), suggesting a pervasive state of T cell stress and dysregulation.

### Distinct B cell profiles are associated with disease severity

**FIGURE 5:**
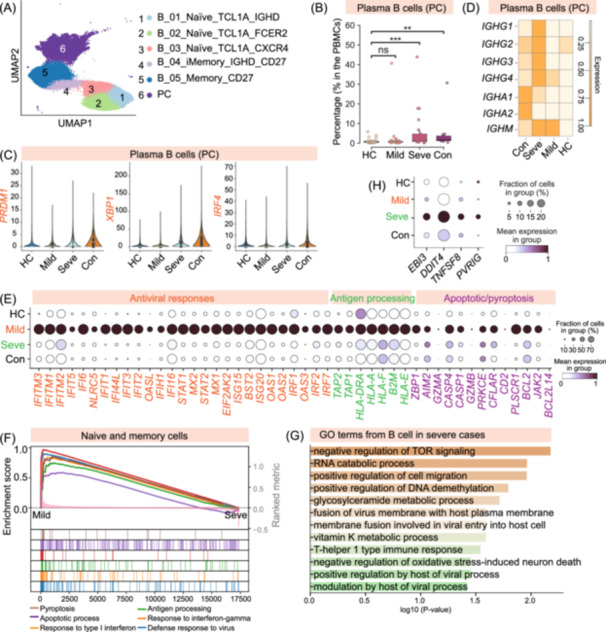
Dysregulated B cell activation and differentiation characterize severe IAV infection. (A) UMAP visualization of B cells and plasma cells, identifying six distinct subsets, including three naïve (B_01–B_03), two memorys (B_04–B_05), and one plasma cell (PC, B_06) cluster. (B) Box plot quantifying the percentage of plasma cells (PCs) within total PBMCs across the four clinical groups (HC, Mild, Seve, Con). (C) Violin plots showing the expression of key plasma cell transcription factors (*PRDM1*, *XBP1*, and *IRF4*) in PCs across the clinical groups. (D) Heatmap showing the average expression of different immunoglobulin heavy chain constant region genes in PCs across the clinical groups, highlighting distinct isotype profiles. (E) Dot plot displaying the expression of representative genes related to antiviral responses, antigen processing, and apoptosis/pyroptosis in naïve and memory B cells (subsets B_01–B_05) across the four clinical groups. (F) Gene Set Enrichment Analysis (GSEA) plot comparing naïve and memory B cells from mild versus severe patients, showing enrichment of antiviral and cell death‐related pathways in the mild group. (G) Bar plot of enriched Gene Ontology (GO) terms for upregulated differentially expressed genes (DEGs) in naïve and memory B cells from severe patients. The *x*‐axis represents the −log10 (*p*‐value). (H) Dot plot showing the expression of key immunomodulatory genes in naïve and memory B cells across clinical groups, highlighting their upregulation in severe disease. For box plots, the center line represents the median, the box limits represent the upper and lower quartiles, and the whiskers extend to 1.5 times the interquartile range. For dot plots, the size of the dot represents the percentage of cells expressing the gene, and the color intensity indicates the average expression level. Asterisks denote statistical significance (*p < 0.05, **p < 0.01, ***p < 0.001; ns, not significant) as determined by Kruskal–Wallis test with Bonferroni correction. IAV, influenza A virus; PBMCs, peripheral blood mononuclear cells; UMAP, Uniform Manifold Approximation and Projection.

To dissect the humoral immune response, we performed unsupervised clustering of B cells, which identified six distinct subsets (Figure 5A, Figure S11A, and Table S2). These subsets included three naïve B cell populations (B_01_Naïve_TCL1A_IGHD, B_02_Naïve_TCL1A_FCER2, and B_03_Naïve_TCL1A_CXCR4), all characterized by expression of *TCL1A* and *IGHD*. However, naïve subsets differed in their expression of markers such as *FCER2* and *CXCR4*, suggesting potential functional or migratory distinctions. We also identified an intermediate memory B cell population (B_04_iMemory_IGHD_CD27) expressing both *IGHD* and *CD27*, a canonical memory B cell cluster (B_05_Memory_CD27) defined by *CD27* expression in the absence of *IGHD*, and a terminally differentiated plasma cell (PC) cluster (Figure 5A). The PC cluster exhibited high expression of classical plasma cell markers, including *MZB1*, *XBP1*, and *CD38* (Figure S11A).

Given their critical role in antibody production, we next focused on the PC compartment. The peripheral PCs highly expressed genes encoding various immunoglobulin constant regions, including *IgA2*, *IgG1*, *IgG2*, *IgG4*, and *IgM* (Figure S11B), indicative of their capacity to secrete diverse antigen‐specific antibodies. Analysis of PC abundance indicated a significant expansion in severe patients compared to mild cases and HC (Figure 5B). Interestingly, these proportions remained significantly higher than HC levels even during the convalescent phase (Figure 5B). This sustained elevation of PCs in severe and convalescent individuals suggests the production of persistently high titers of IAV‐specific antibodies, a phenomenon also observed in severe COVID‐19 [17]. Consistent with heightened antibody production, key transcription factors essential for plasma cell differentiation and function, including *PRDM1*, *XBP1*, and *IRF4*, were significantly upregulated in PCs from severe patients relative to those from mild patients and HC (Figure 5C). These transcription factors reached their highest expression levels in convalescent individuals, suggesting ongoing antibody synthesis and the potential establishment of long‐lived plasma cell responses during recovery. Further examination of immunoglobulin isotype expression within PCs indicated two distinct patterns: IGHM (encoding IgM) was more prominent in PCs from mild patients, potentially reflecting an earlier stage of the humoral response or a specific IgM‐mediated protective mechanism (Figure 5D). In contrast, *IGHA1* and *IGHA2* (encoding *IgA1* and *IgA2*) were most highly expressed in PCs from convalescent individuals (Figure 5D), suggesting a shift towards mucosal immunity or a specific role for IgA in long‐term protection. Together, these data highlight a pronounced and sustained plasma cell response, particularly in severe disease, characterized by dynamic changes in key regulatory transcription factors and immunoglobulin isotype profiles across different disease stages.

Examination of naïve and memory B cell precursors indicated an unexpected expansion. Instead of the anticipated naïve B cell depletion, the proportions of all three naïve and both memory B cell subsets were elevated in severe and convalescent patients compared with mild patients (Figure 1D). This broad increase suggests a systemic perturbation during severe disease, such as enhanced B cell mobilization or survival, distinct from canonical antigen‐driven consumption. DGE analysis of combined naïve and memory B cells (B_01‐B_05) across patient groups indicated transcriptional landscapes that diverged with disease severity (Figure S11C and Tables S15–17). Notably, B cells in mild cases uniquely upregulated a diverse repertoire of 345 genes, enriched for functions including antigen presentation (e.g., representative genes include *HLA‐DR*, *TAP2*), antiviral responses (e.g., *IFITM3*, *ISG20*), and programmed cell death pathways (*CASP1*/*4*, *BCL2L14*, *JAK2*), as confirmed by GO/GSEA (Figure 5E,F, Figure S11D, and Tables S15–17). Indeed, these biological pathways (e.g., defense response to virus) were systematically elevated across all B cell subsets, specifically in mild patients (Figure S11E). Herein, these findings suggest a multifaceted and robust B‐cell response in mild disease.

In contrast, naïve and memory B cells from severe cases exhibited a narrower transcriptional signature, uniquely upregulating only 64 genes (Figure S11C). GO analysis indicated that this signature was enriched for pathways of immune regulation, cellular stress, and host‐virus interactions (Figure 5G). For instance, enriched terms included “T‐helper 1 type immune response” and “negative regulation of TOR signaling,” pointing to a state of active immunomodulation rather than broad effector function. This modulatory profile was underscored by the upregulation of key regulatory genes, included *EBI3*, a component of the suppressive cytokines *IL‐27/IL‐35*, and *DDIT4*, an inhibitor of the mTOR pathway essential for cell growth (Figure 5H). Furthermore, the immune checkpoint molecule *PVRIG* and the T‐cell co‐stimulatory ligand *TNFSF8* (*CD153*) were also elevated (Figure 5H). The concurrent expression of these genes indicates that B cells in severe patients, while numerically expanded, are driven towards a specialized state of functional constraint and regulatory crosstalk. This focused, immunomodulatory signature contrasts sharply with the multifaceted response in mild disease, implying that B cells in severe cases may contribute to immune dysregulation.

### Dichotomous myeloid responses define disease severity and outcome

**FIGURE 6:**
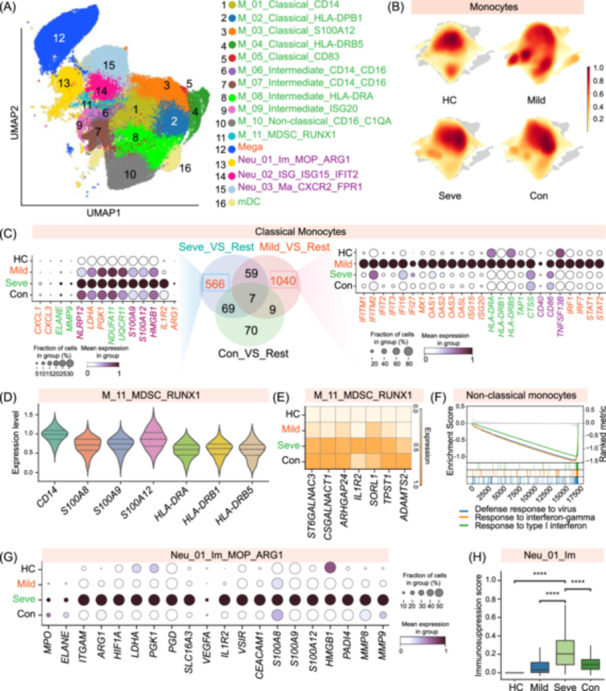
A monocyte‐to‐neutrophil shift and myeloid dysfunction define severe IAV infection. (A) Uniform Manifold Approximation and Projection (UMAP) visualization of 16 myeloid cell subsets identified by subclustering. (B) UMAP density plots showing the distribution of monocytes in each of the four clinical groups, highlighting the polyclonal activation in mild disease. The color scale indicates cell density. (C) Venn diagram showing the overlap of upregulated differentially expressed genes (DEGs) in classical monocytes (M_01‐M_05) from Severe, Mild, and Convalescent patients compared to the rest (Middle panels). Dot plots displaying the expression of representative genes from the Mild‐specific protective signature (left) and the Severe‐specific pathogenic signature (right). (D) Violin plots showing the expression of canonical mMDSC markers (*CD14*, *S100A8/9/12*) and the hallmark low expression of MHC class II genes (*HLA‐DRA/DRB1/DRB5*) in the M_11_MDSC_RUNX1 subset. (E) Heatmap showing the expression of uniquely upregulated pathogenic genes in the M_11_MDSC_RUNX1 subset from severe patients. (F) GSEA plot for non‐classical monocytes (M_10), showing the negative enrichment of antiviral and interferon response pathways in severe compared to mild patients. (G) Dot plot displaying the expression of genes associated with the dual pathogenic signature (immunosuppression and inflammation) of immature neutrophils (Neu_01_Im_MOP_ARG1) across the four clinical groups. (H) Box plot comparing the immunosuppression module score for the Neu_01_Im_MOP_ARG1 subset across the clinical groups. For dot plots, the size of the dot represents the percentage of cells expressing the gene, and the color intensity indicates the average expression level. For the box plot, the center line represents the median, the box limits represent the upper and lower quartiles, and the whiskers extend to 1.5 times the interquartile range. Asterisks denote statistical significance (****p < 0.0001) as determined by Kruskal–Wallis test with Bonferroni correction. GSEA, Gene Set Enrichment Analysis; IAV, influenza A virus.

To dissect myeloid heterogeneity in IAV infection, we re‐clustered all myeloid cells, which resolved into 16 distinct subpopulations (Figure 6A, Figure S12A). These include 11 monocyte, three neutrophil, one mDC, and one megakaryocyte (Mega) cluster, providing a granular map of myeloid compartment. The monocyte subsets were stratified into five classical, four intermediate, one non‐classical, and one myeloid‐derived suppressor cell (MDSC)‐like subset (Figure S12B,C). Multiple classical and intermediate subsets exhibited activated phenotypes (e.g., expressing *HLA‐DPB1*, *HLA‐DRB5*, *CD83*, *HLA‐DRA*, or *ISGs*) (Figure S12A). The non‐classical subset was uniquely defined by high *CD16* and complement gene expression (*C1QA/B/C*), and we also identified a MDSC‐like population with a suppressive *S100A8*/*A9* ^+^ *HLA‐DR* ^−/low^ signature (Figure S12A). Neutrophils were resolved into three functional states, including immature (*MPO* ^+^), ISG‐high (*ISG15* ^+^), and mature (*CXCR2* ^+^) populations (Figure S12A). *R*_O/E_ analysis indicated a dichotomous myeloid landscape that distinguished disease severity (Figure 1D). Mild patients were characterized by a notable enrichment of all 11 monocyte subtypes, suggesting a robust innate response. In contrast, severe disease was marked by an expansion of all three neutrophil subsets, implicating a dysregulated granulocytic response consistent with the observed inflammatory storm. This fundamental myeloid divergence, a monocyte‐centric response in mild disease versus a neutrophil‐centric one in severe cases, defines a key immunological axis determining influenza outcome.

Next, we first focused on monocytes, given their abundance and expansion in mild disease. This divergence was clearly visualized in UMAP density plots (Figure 6B). Mild disease was uniquely characterized by multiple, distinct high‐density regions, reflecting broad polyclonal activation, whereas severe disease drove a more focused cellular redistribution. To dissect the connectivity underlying monocyte states, PAGA analysis (Partition‐based graph abstraction) indicated notably different network topologies between mild and severe diseases (Figure S12B). In mild cases, monocytes formed a highly interconnected and complex network, indicative of a coordinated and multifaceted response involving all subsets. In contrast, the PAGA network was profoundly rewired in severe disease into a restricted, inflammatory circuit that was depleted of the antiviral M_09_Intermediate_ISG20 subset (Figures S12B, Figure 1D). To identify the cellular architects of these divergent networks, we defined hub cells as the top 5% of monocytes with the highest PAGA connectivity (Figure S13A). In mild cases, the network was anchored by a trio of activated classical (M_04_Classical_HLA‐DRB5), antiviral intermediate (M_09_Intermediate_ISG20), and M_07_Intermediate_CD14_CD16 monocytes (Figure S13B). Crucially, this protective axis collapsed in severe disease, with the network re‐centering on a distinct set of hubs, including M_08_intermediate_HLA‐DRA and the pro‐inflammatory M_03_Classical_S100A12 subset (Figure S13B), highlighting a profound functional shift in the monocyte command structure.

Classical monocytes (M_01/02/03/04/05) adopted starkly divergent transcriptional programs that were associated with disease outcome (Figure S13A,B). In mild cases, these subsets orchestrated a protective response, upregulating a broad number of antiviral genes (e.g., *IFITM1*, *MX1*, *OAS1/2/3*, *ISG15*), antigen presentation machinery (e.g., *HLA‐DRA/B1/B5*, *TAP1*, *CTSS*), adaptive immunity initiators (*CD40*, *CD86*, *TNFSF13B*), and key antiviral transcription factors (e.g., *IRF1*, *IRF7*, *STAT1*, *STAT2*) (Figure 6C and Tables S18–20). GSEA confirmed this protective phenotype, with enrichment for “interferon response,” “defense response to virus,” and “innate immune response” pathways (Figure S13C). In severe patients, this protective program was supplanted by a pathogenic signature, characterized by the expression of inflammatory mediators (e.g., *CXCL1*, *CXCL3*), tissue‐damaging proteases (e.g., *ELANE*, *MMP9*), inflammasome components (e.g., *NLRP12*), DAMPs (e.g., *S100A9*, *S100A12*, *HMGB1*), and immunosuppressive molecules (e.g., *IL1R2*, *ARG1*) (Figure 6C). This pathogenic rewiring was coupled with profound metabolic reprogramming, marked by heightened glycolysis (e.g., *LDHA*, *PGK1*) and impaired mitochondrial respiration (e.g., *NDUFA11*, *UQCR11*) (Figure 6E). GSEA analysis also indicated the enrichment for “neutrophil activation” and “glycolytic process” in severe disease, implicating a cellular state that actively fuels immunopathology (Figure S13D). This functional reprogramming of classical monocytes from a protective to a pathogenic state represents a critical checkpoint in influenza progression. Particularly, this disease‐dependent functional polarization was a defining feature across the monocyte continuum. Mirroring the classical monocytes, intermediate monocytes also exhibited a functional dichotomy, shifting from a protective, interferon‐driven state in mild disease to a pro‐inflammatory, metabolically stressed phenotype in severe patients (Figure S14A–C).

Among the monocyte subsets, we identified a monocytic MDSC (mMDSC) subtype (M_11_MDSC_RUNX1) (Figure 6A). These cells exhibited the canonical mMDSC *CD14*⁺*HLA‐DR* ^−^/^low^ phenotype and high expression of calprotectin (*S100A8/9/12*), consistent with a phenotype previously linked to potent T‐cell‐suppressive capacity (Figure 6D) [17]. To validate this transcriptomic finding at the protein level, we performed flow cytometry on PBMCs from severe patients, which confirmed the significant presence of a *CD14* ^+^ *HLA‐DR* ^−/low^ population, corroborating the identity of this immunosuppressive subset (Figure S12C). Particularly, mMDSC from severe patients upregulated a unique set of pathogenic genes (Figure 6E). For instance, mMDSCs upregulated genes related to aberrant cell adhesion and extracellular matrix (ECM) remodeling, including *SORL1*, *ADAMTS2*, and *TPST1*. These cells also exhibited increased expression of genes implicated in dysregulated signaling, such as the IL‐1‐neutralizing decoy receptor *IL1R2* and the signaling scaffold *ARHGAP24*. Moreover, the elevated expression of glycosyltransferases *ST6GALNAC3* and *CSGALNACT1* suggested that mMDSC may alter the local glycan landscape, a mechanism increasingly recognized for its role in immune modulation. These data indicate that during severe infection, mMDSCs express a multifaceted transcriptional program that likely contributes to T cell suppression and a pro‐pathogenic tissue microenvironment.

We also identified a non‐classical monocyte subset (M_10_Non‐classical_CD16_C1QA), defined by a CD16⁺CD14^−^ phenotype and high *C1QA/B/C* expression (Figure 6A, Figure S12A). These monocytes were the primary source of peripheral C1 complement components (Figure S14D). In severe patients, non‐classical monocytes upregulated a transcriptional program related to scavenger receptor signaling, phagocytosis, and inflammation. This included upregulation of scavenger receptors (*CD163*, *MRC1*, *STAB1*), phagocytic genes (*ITGAM*, *FCGRT*, *LYZ*), and actin‐remodeling proteins (*VIM*, *PFN1*, *CORO1A*) crucial for cellular uptake (Figure S15A). In addition, non‐classical monocytes had a hyperinflammatory phenotype, marked by elevated expression of alarmins (*S100A8*/*A9*/*A12*), cathepsins (*CTSB*/*D*/*H*), and leukotriene biosynthesis genes (*ALOX5*, *ALOX5AP*, *LTA4H*). This state was accompanied by metabolic rewiring towards glycolysis (*LDHB*, *PKM*, *TPI1*) and heightened mitochondrial stress, indicated by upregulation of numerous genes encoding mitochondrial ribosomal proteins and subunits of respiratory chain complexes I–V (e.g., *NDUF*, *COX*, *UQCR*, *ATP5* family members) (Figure S15A). GSEA analysis further indicated a profound loss of their protective functions in severe disease, with significant negative enrichment of antiviral pathways, including “defense response to virus,” “response to type I interferon,” and “response to interferon‐gamma” (Figure 6F). In contrast, positively enriched pathways, such as “inner mitochondrial membrane organization,” and “fatty acid biosynthesis,” pointed to cellular stress and metabolic dysregulation (Figure S15B). These results highlighted that the pathogenic state of non‐classical monocytes in severe cases is defined not by a simple hyperinflammatory switch, but by a failure to sustain antiviral immunity.

Our analysis identified three neutrophil subsets notably enriched in patients with severe cases (Figure 1D). Among these, the immature neutrophil population (Neu_01_Im) was more notably expanded in severe and convalescent (Figure 1D). Given that this subset has been previously implicated in severe infections and exhibited a dual pathogenic signature [33], we focused on elucidating its potential pathogenic mechanisms. The Neu_01_Im subset highly expressed canonical immaturity markers (*MPO*, *ELANE*, *ITGAM*) while also being transcriptionally programmed for potent immunosuppression (Figure S15C), as evidenced by high *ARG1* expression (Figure S15D). This phenotype validated their identity as granulocytic MDSCs (G‐MDSCs). A defining characteristic of G‐MDSCs was a profound metabolic shift towards hypoxia‐driven glycolysis, a known hallmark of immunosuppressive myeloid cells. This reprogramming was orchestrated by the significant upregulation of the master regulator *HIF1A* and its downstream targets, including key glycolytic enzymes (*LDHA*, *PGK1*, *PGD*), the lactate transporter *SLC16A3*, and the immunosuppressive factor *VEGFA* (Figure S15E). Further contributing to the immunosuppressive function, G‐MDSCs also expressed high levels of *ARG1*, an enzyme that depletes l‐arginine from the microenvironment, thereby inhibiting T cell proliferation and function (Figure S15E). In addition to this metabolic mechanism, the G‐MDSCs displayed a repertoire of inhibitory molecules, including the IL‐1 decoy receptor *IL1R2*, the checkpoint inhibitor VSIR (VISTA), and *CEACAM1* (Figure S15E). Particularly, the suppressive profile was augmented by high expression of damage‐associated molecular patterns (*S100A8*/*9*/*12* and *HMGB1*) and proteases (*PADI4* and *MMP8*/*9*), which may foster a suppressive environment via mechanisms potentially including NETosis, given the high expression of the rate‐limiting enzyme *PADI4*. Consistently, the genes encoding these factors were notably upregulated within the Neu_01_Im subset from severe patients, suggesting this cellular program drives pathogenesis in severe cases. Indeed, an integrated immunosuppression score indicated that the Neu_01_Im population from severe cases constituted the most potent suppressive state compared to other IAV disease conditions (Figure 6H). These results position these emergency‐mobilized, immature neutrophils as an important driver of the T cell dysfunction characteristic of severe patients.

## DISCUSSION

IAV remains a major global public health threat, causing substantial annual morbidity and mortality through seasonal epidemics and posing a persistent pandemic risk [34]. Despite the severe clinical burden, ranging from mild illness to life‐threatening viral pneumonia and ARDS, the immune determinants underlying these divergent outcomes remain poorly defined [4, 35]. To investigate this, we generated a large‐scale, single‐cell transcriptomic atlas of the peripheral immune response during human IAV infection, providing high‐resolution insight into its cellular and molecular dynamics (Figure 1). This atlas profiled over 612,010 cells from 97 individuals, resolves 44 distinct immune subsets and importantly captured granulocytic populations that are typically lost during standard PBMC processing [26], thereby highlighting its fidelity in reflecting systemic inflammation. Analysis of the dataset indicates a distinct immunological bifurcation that correlates with disease severity: a coordinated, monocyte‐dominated protective response in mild illness versus a dysregulated, neutrophil‐ and MDSC‐dominated pathological response in severe disease. Severe disease was characterized by profound lymphopenia, with a marked depletion of T and NK cell populations that persists into convalescence, indicating long‐lasting immune dysregulation. In contrast, mild illness featured a monocyte‐dominant response, evidenced by the expansion of specific activated and interferon‐stimulated monocyte subsets. This fundamental divergence (a T/NK cell deficit vs. a controlled monocyte expansion) establishes an immunological framework for IAV pathogenesis and a foundation for investigating the cellular states and pathways that determine protective versus pathogenic outcomes.

Our analysis indicated that the hyperinflammatory state in severe IAV infection is driven primarily by specific hyperinflammatory monocyte and neutrophil subsets rather than a diffuse response (Figure 2). Notably, this pronounced myeloid dysregulation was a consistent feature across severe patients, persisting against the background of standard‐of‐care interventions such as antiviral therapy and respiratory support. The intensified inflammatory milieu likely arises from heightened per‐cell transcriptional activity in these subsets rather than cellular expansion. Specifically, we identified the *S100A8/A9/A12* alarmin family as key molecular drivers of the hyperinflammatory response. To confirm these observations, we validated key transcriptomic findings, such as the hyperinflammatory S100 signature and immune cell abundance changes, using plasma ELISA and clinical data. These orthogonal data confirm the robustness of our computational analysis, supporting the broader functional inferences drawn from this atlas. This finding was supported by elevated plasma S100 concentrations in severe patients. Moreover, the co‐expression of their cognate receptors, including *TLR4* and *RAGE*, on these same myeloid cells indicates a potent autocrine and paracrine feedback loop that drives a self‐amplifying inflammatory cascade. Although profiled in the periphery, these hyperinflammatory myeloid subsets likely represent the active “cellular reinforcements” mobilized from the bone marrow to the infected lung. Their pathogenic programming in the blood suggests that the systemic circulation acts as a mirror of the respiratory microenvironment, reflecting “spillover” signals from the site of infection. This S100‐driven myeloid hyperactivation suggests a mechanism of convergent immunopathology shared with other severe respiratory infections, including COVID‐19 and bacterial pneumonia [15, 21]. However, distinct cellular dynamics distinguish these conditions. While severe COVID‐19 is frequently marked by a profound expansion of classical monocytes and depletion of non‐classical monocytes [15], our IAV atlas indicates a more complex rewiring where specific hyperinflammatory subsets drive pathology amidst a broader myeloid dysregulation. Similarly, while bacterial pneumonia typically elicits a massive, acute neutrophilic leukocytosis [21], the severe IAV response is characterized by the specific mobilization of immature, immunosuppressive neutrophil subsets (G‐MDSCs) alongside the inflammatory storm. This suggests that while the molecular “alarmins” (*S100* proteins) are shared, the cellular vehicles and adaptive immune consequences are pathogen‐specific. Therapeutically, identifying the *S100–TLR4/RAGE* axis as a key driver of severity highlights actionable targets for drug repurposing. For instance, small‐molecule inhibitors of *S100A9* (e.g., Paquinimod) and *RAGE* antagonists (e.g., Azeliragon), which have shown promise in autoimmune and chronic inflammatory diseases [36], warrant investigation as host‐directed therapies to dampen the cytokine storm in severe influenza without abrogating viral clearance.

The T cell response to IAV infection is similarly divergent, correlating with disease severity. In mild disease, a functionally balanced T cell response orchestrates protective immunity. CD8^+^ T cells co‐express markers of both high cytotoxicity and exhaustion (Figure 3), a transcriptional signature characteristic of a mature, controlled response that can clear IAV‐infected cells while limiting immunopathology. This response is bolstered by a multifaceted CD4^+^ T cell compartment, including cytotoxic effectors with robust interferon‐stimulated programs and an expanded Th2‐like population expressing *IL‐4* and *IL‐21* to promote humoral immunity (Figure 4). This profile defines an effective, self‐regulating adaptive response. In contrast, severe disease is marked by a profound collapse of T cell function. T cells from severe patients exhibit transcriptional signatures of terminal dysfunction, including severe metabolic stress, mitochondrial failure, and apoptosis (Figures 3 and 4), which provide a cellular basis for the observed lymphopenia and directly impair viral control. The transcriptional evidence of functional paralysis in T cells appears to be exacerbated by a pathogenic rewiring of Tregs. Notably, eTregs in severe cases undergo a pathogenic conversion, abandoning their canonical suppressive function to adopt a pro‐inflammatory phenotype and express damage‐associated molecules (e.g., *S100A4* and *HMGB1*). The convergence of pathogenic Treg activity, depletion of supportive Th2‐like cells, and the metabolic demise of T cells establishes a vicious cycle of immunopathology. Furthermore, we hypothesize that a synergistic crosstalk likely exists between these pathogenic Tregs and the expanded MDSCs, forming a positive feedback loop that deepens T cell paralysis and prevents immune resolution. Thus, these findings redefine T cell failure in severe IAV infection not as passive dysregulation, but as an active collapse driven by metabolic crisis and the pathogenic conversion of regulatory cells.

This functional dichotomy also extends to the humoral immune system, where divergent B cell landscapes are associated with disease outcome. The B cell compartment from mild cases is characterized by a multifaceted protective response. In addition to robust IgM‐skewed plasma cell differentiation, naïve and memory B cells exhibit transcriptional signatures indicative of broad antiviral activity, including the upregulation of interferon‐stimulated genes and genes involved in antigen presentation (Figure 5). This suggests that B cells, beyond their role as antibody precursors, contribute directly to orchestrating a coordinated antiviral state that reinforces the T cell response. However, the humoral response in severe patients was profoundly dysregulated. Despite a marked expansion of plasma cells, their precursor B cells exhibited a distinct immunomodulatory profile, upregulating genes such as *EBI3* (a component of IL‐35) and the immune checkpoint molecule *PVRIG* (Figure 5). The transcriptional state of B cells in severe disease suggests a shift towards an immunosuppressive or functionally constrained phenotype relative to that in mild illness. This functional uncoupling within the B cell compartment, where substantial plasma cell expansion occurs alongside the suppressive programming of their precursors, likely contributes to the systemic immune dysregulation and exacerbate the observed T cell failure.

Our findings further indicate a profound polarization in the peripheral myeloid response that underpins divergent clinical trajectories in IAV infection. Mild illness is characterized by a protective, monocyte‐centric program, featuring a coordinated expansion of monocyte subsets (Figure 1). Particularly, these monocytes establish a robust antiviral state, marked by potent interferon signaling and enhanced antigen presentation capacity, thereby facilitating viral clearance and initiating adaptive immunity (Figure 6). Severe illness, however, is characterized by a functional shift towards a pathogenic, monocyte‐ and neutrophil‐driven state combining hyperinflammation with immunosuppression. This pathogenic state is defined by the emergence of hyperinflammatory monocytes that fuel hyperinflammation via the *S100–TLR4/RAGE* axis, coupled with the expansion of both granulocytic and monocytic MDSCs (Figures 2 and 6). These MDSCs are predicted to establish an immunosuppressive microenvironment, mediating T cell suppression through metabolic reprogramming towards hypoxia‐inducible glycolysis. While functional co‐culture assays were limited by sample availability, the immunosuppressive role of these subsets is well‐supported by their distinct phenotypes (e.g., *ARG1* ^+^neutrophils, *HLA‐DR* ^−/Low^ monocytes) and extensive prior characterization in similar inflammatory contexts. Previous studies have firmly established that the expansion of MDSCs is a hallmark of immune paralysis in severe COVID‐19 and sepsis [28, 37, 38], where they directly inhibit T cell proliferation. Our findings extend this paradigm to influenza, identifying MDSCs as a critical, yet previously underappreciated, driver of severe IAV pathogenesis. This polarization of the myeloid response, from protective antiviral immunity to a deleterious state of combined hyperinflammation and profound immunosuppression, likely represents a critical bifurcation point in influenza pathogenesis. Therefore, therapeutic strategies aimed at reprogramming these myeloid compartments (e.g., by inhibiting MDSC function or restoring protective monocyte programs), may hold significant promise for ameliorating severe influenza. Furthermore, from a diagnostic perspective, the S100A8/9/12 signature may offer a feasible tool for clinical stratification. These proteins can be rapidly quantified via standard, low‐cost plasma assays (ELISA), providing a high‐sensitivity biomarker for identifying patients at risk of hyperinflammation.

Addressing the direction of causality between immune dysregulation and disease severity is critical. We acknowledge that our cross‐sectional design identifies associations rather than proving temporal causality. While our cross‐sectional analysis inherently captures a snapshot of the immune state, the specific molecular programs we identified suggest that these cellular responses are not merely passive consequences of high viral load, but active drivers of pathogenesis forming a maladaptive feed‐forward loop. Specifically, the co‐expression of *S100* alarmins and their receptors (*TLR4*/*RAGE*) on myeloid cells creates a self‐sustaining inflammatory circuit that likely perpetuates tissue damage independently of the viral trigger. Concurrently, the metabolic collapse and suppressive programming of T cells prevent effective viral clearance, thereby prolonging the infection and exacerbating severity. Thus, we propose that severe influenza is driven by a “double hit”: the active generation of immunopathology by hyperinflammatory myeloid cells and the simultaneous failure of adaptive resolution mechanisms.

While this atlas provides a comprehensive transcriptomic blueprint of the peripheral immune response to IAV, several limitations exist. First, we focused on peripheral blood rather than the lung microenvironment or bronchoalveolar lavage fluidF), as ethical and safety constraints precluded invasive sampling in severe patients. However, peripheral blood serves as a validated systemic window for characterizing immunopathology in severe infections [15, 21, 39]. Second, while we validated key inflammatory markers and cell proportions via ELISA and clinical data, functional states (e.g., T cell exhaustion, MDSC suppression) are inferred from transcriptomics and warrant confirmation through ex vivo functional assays. However, the consistency of our findings with established molecular mechanisms (e.g., *S100*‐*TLR4* axis) and clinical protein‐level data supports the robustness of these inferences. Third, although strong pathogenic signatures in severe disease likely override demographic confounders, we did not analytically correct for factors such as age. Advanced age is the primary risk factor for severe influenza; thus, treating it as a technical covariate to be removed could obscure the biological reality of host susceptibility. Importantly, the resolution of pathogenic signatures (e.g., S100 storm) in the age‐matched convalescent group confirms that these immune alterations are driven by the acute infection rather than patient age. Fourth, utilizing healthy control data from previous studies may introduce technical bias, despite rigorous batch correction. However, the distinct biological stratification of concurrently recruited patient groups suggests that disease‐specific signals override technical variance. Finally, our cross‐sectional design limits temporal resolution. While we employed trajectory inference algorithms to reconstruct cellular developmental paths, these represent inferred pseudotemporal states rather than longitudinally observed progression within individuals. Future longitudinal multi‐omic studies tracking patients from symptom onset to recovery are needed to validate the precise kinetics of these immune transitions.

## CONCLUSION

Our large‐scale single‐cell atlas provides a high‐resolution immunological map of human IAV infection, indicating the dichotomy of peripheral immune responses associated with clinical severity. Protective immunity in mild illness relies on a coordinated myeloid, T and B cell response, characterized by robust antiviral signaling and balanced effector functions. Severe disease is driven by a dysregulated myeloid circuit generating hyperinflammation and immunosuppression, with concomitant systemic T and B cell dysfunction exacerbating pathology. Specifically, we conclude that this immune dichotomy is driven by the functional differentiation of monocytes and neutrophils, where the *S100A8/9/12*–*TLR4/RAGE* axis and MDSCs represent the key pathogenic targets. Collectively, our atlas elucidated key mechanisms of IAV host‐immune pathogenesis and provides a foundational resource for designing targeted immunotherapies to ameliorate severe IAV infection.

## METHODS

### Ethical approval

Our study was approved by the Ethics Committee of the Chinese PLA General Hospital (approval no. 2022113030901836) and adhered to the tenets of the Declaration of Helsinki. Prior to inclusion in the study, all participants or their legal guardians provided written informed consent.

### Study design and participants

A total of 97 individuals were included in the final integrative analysis. This cohort was comprised of 61 newly recruited patients with confirmed IAV infection and 36 healthy controls (HC) (Table S1 *)*. To maximize resource efficiency and utilize high‐quality existing data, the raw sequencing data for the 36 HC samples were retrieved from our group's previously published datasets and re‐processed uniformly with the IAV samples [21, 40]. The patient cohort was stratified into mild (*n* = 30), severe (*n* = 21), and convalescent (*n* = 10) groups from severe disease. Viral subtyping confirmed that all patients were infected with the Influenza A (H1N1) subtype, reflecting the dominant circulating strain during the study period. All participants were recruited from seven hospitals in Beijing between December 2024 to January 2025.

To ensure the transcriptomic signatures reflected a pure IAV immune response, we applied rigorous exclusion criteria regarding co‐infections. All patients underwent comprehensive microbiological screening upon admission, including multiplex PCR for a panel of common respiratory viruses (Influenza B, Adenovirus, RSV, Parainfluenza) and atypical pathogens (*Mycoplasma pneumoniae*, *Chlamydia pneumoniae*, *Legionella pneumophila*), as well as standard bacterial and fungal cultures. Only patients testing positive exclusively for IAV and negative for all other co‐pathogens were enrolled. Furthermore, we acknowledge that the severe patient group was significantly older than the mild group (Figure S1E). This demographic distribution reflects the natural epidemiology of influenza, where advanced age is the primary risk factor for the development of severe viral pneumonia [41]. Consistent with prior single‐cell atlases of severe infectious diseases (e.g., COVID‐19 and tuberculosis) [15, 22], our analysis treats disease severity as the primary driver of the acute immune response, although age contributes to the host's susceptibility to this severe state.

In accordance with guidelines established by the Infectious Diseases Society of America and the Chinese national protocol for influenza diagnosis and treatment (2025 Edition), severe IAV pneumonia was defined by the presence of one or more of the following criteria: (1) acute respiratory failure requiring mechanical ventilation; (2) shock requiring vasopressor support; (3) acute necrotizing encephalopathy; or (4) multiple organ dysfunction necessitating admission to an intensive care unit. Individuals were excluded from the study based on the following criteria: (1) under 18 years of age; (2) pneumonia caused by co‐infection with other viral or bacterial pathogens; (3) a known history of autoimmune disease; (4) clinical immunosuppression (including corticosteroid or chemotherapy treatment, organ transplantation, hematologic malignancy, or HIV infection with a CD4^+^T‐cell count < 200 cells/µL); or (5) significant pre‐existing respiratory comorbidities, such as asthma, chronic obstructive pulmonary disease, cystic fibrosis, or bronchiectasis.

### Single‐cell RNA sequencing and data analysis

Peripheral blood mononuclear cells (PBMCs) were isolated from blood samples collected within 2 h of diagnosis from patients with mild or severe IAV disease. Cell viability was immediately assessed using a Countstar cell viability assay (ALIT Life Science, Shanghai, China), only samples exceeding 90% viability were processed for downstream analysis [40]. Single‐cell RNA sequencing libraries were subsequently prepared using the Chromium Single Cell 5ʹ v2 Kit (10×Genomics, PN‐1000263, Pleasanton, CA) following the manufacturer's protocol. The resulting libraries were sequenced on an Illumina NovaSeq. 6000 platform (San Diego, CA) to generate 2 × 150 bp paired‐end reads.

The scRNA‐seq data analysis pipeline was adapted from previously described methods [17, 22]. Raw sequencing data were processed using kallisto/bustools (v0.24.4) to generate gene expression matrices for each of the 97 samples, and these matrices were then concatenated using anndata (v0.7.6). Following quality control, wherein low‐quality cells and doublets were removed with Scanpy (v1.9.2). Specifically, low‐quality cells were filtered out if they detected fewer than 200 genes, fewer than 500 UMIs, or had a mitochondrial gene percentage greater than 15%. Subsequently, the data were normalized to 10,000 reads per cell. A consensus set of 1500 highly variable genes (HVGs) was selected based on recovery rates across samples, after excluding ribosomal, mitochondrial, and immunoglobulin genes [20]. Genes encoding ribosomal proteins, mitochondrial components, and immunoglobulins were systematically excluded from this list to prevent clustering bias. Principal component analysis (PCA) was performed on this HVG set. To integrate the data while preserving biological heterogeneity across the large cohort (>600,000 cells), we utilized the Harmony algorithm within the Scanpy (Python) environment.

We selected the Python‐based workflow over R‐based alternatives (e.g., Seurat's FindIntegrationAnchors) primarily for its superior computational efficiency and scalability when processing large‐scale datasets. Furthermore, Harmony is particularly well‐suited for integrating datasets generated with a uniform chemistry (10×Genomics 5' v2) where batch effects are primarily technical (donor‐specific), as it corrects the low‐dimensional embedding without aggressively altering the underlying gene expression values, thereby preserving rare cell populations.

Batch correction was performed on the top 20 principal components using Scanpy's external.pp.harmony_integrate function [42, 43]. To account for technical variation, “Sample_ID” and “Sequencing_Batch” were treated as covariates. The diversity penalty parameters were set to theta = 2.5 for Sample_ID and theta = 1.5 for Sequencing_Batch to balance effective mixing with the retention of biological structure [43, 44]. Convergence was monitored to ensure stable integration. Finally, cell clustering was performed on the corrected PCA matrix using the Louvain algorithm, and marker genes for each cluster were identified using the rank_genes_groups function [45].

### Cell clustering and annotations

Cell populations were identified through an iterative Louvain clustering approach. An initial clustering round (resolution = 2.0) resolved 12 major cell lineages: B cells, plasma cells (PCs), CD4^+^ T cells, CD8^+^ T cells, MAIT cells, γδ T cells, NK cells, monocytes, neutrophils, mDCs, megakaryocytes, and red blood cells. The identity of each subcluster was determined based on the expression of canonical marker genes. These assignments were further refined by examining cluster‐defining differentially expressed genes (DEGs) identified using Scanpy's rank_genes_groups function.

To investigate the developmental relationships between immune cell states, we performed trajectory inference using PAGA [46]. DEGs between cell clusters or disease conditions were identified using a Wilcoxon rank‐sum test (rank_genes_groups, use_raw = True), with significance defined by a Benjamini–Hochberg adjusted *p*‐value < 0.01 and a fold‐change threshold > 1.5 to correct for multiple testing. Finally, potential ligand‐receptor interactions were inferred using CellPhoneDB (alpha = 0.01, *p* < 0.01).

### Identifying changes in immune cell proportion

Alterations in the proportional representation of immune cell populations across disease conditions were evaluated using several statistical approaches. Differences in the relative abundance of each population were first assessed with a Kruskal–Wallis test, followed by Bonferroni correction for multiple comparisons. Subsequently, multivariate analysis of variance was employed to model the contributions of disease stage and potential interaction terms [22, 47]. The enrichment of specific cell populations within each disease state was then quantified by calculating the ratio of observed to expected (RO/E) cell counts [22].

### Determining cell state scores

To assess shifts in cell‐type‐specific functional states across disease phases, we implemented a gene set scoring analysis. Signature gene modules representing key biological processes, including proinflammatory signaling, cytotoxicity, cellular exhaustion, and regulatory functions, were curated from previously published studies [30, 40]. To quantify cell‐type‐specific functional states, we calculated “Cytokine Scores” and “Inflammatory Scores” using established gene signatures [15, 21, 39]. These gene lists were curated from the validated immunological literature (Table S3). Scores were computed on a per‐cell basis using the score_genes function in Scanpy (v1.9.2). This algorithm calculates the average expression of the genes in the target set and subtracts the average expression of a reference set of randomly sampled genes matched for expression level. This approach effectively normalizes for cellular sequencing depth and technical variation. Comparisons of these scores across clinical groups (HC, Mild, Severe, Convalescent) were assessed using the Kruskal–Wallis test with Bonferroni correction for multiple comparisons. A score for each module was calculated on a per‐cell basis using Scanpy's score_genes function, which computes the average expression of genes in the set, normalized against a background of randomly selected genes. Comparisons of these scores across clinical groups (HC, Mild, Severe, and Convalescent) were assessed using the Kruskal–Wallis test. If the omnibus test was significant, post hoc pairwise comparisons were performed using Dunn's test. To control the family‐wise error rate, *P*‐values were adjusted using the Bonferroni correction method, with the adjustment scope covering all possible pairwise combinations (*m* = 6) within each independent test. For GSEA, we utilized the GO Biological Process (BP) gene sets obtained from the Molecular Signatures Database (MSigDB).

### Plasma cytokine quantification

Plasma cytokine levels were quantified using the Th1/Th2 34‐plex human ProcartaPlex kit (Thermo Fisher Scientific) according to the manufacturer's instructions [48]. To ensure quantitative reliability, samples were analyzed in duplicate, with the lower limit of quantitation for individual analytes following manufacturer specifications. Intra‐assay and inter‐assay coefficients of variation were maintained below 10% and 15%, respectively.

### Statistical analysis

Statistical analyses and data visualization were performed using Python and R. Correlation analyses were performed using Spearman's rank correlation coefficient unless otherwise noted. Statistical significance is indicated in all figures as follows: ns, not significant (*p* > 0.05); *p < 0.05; **p < 0.01; ***p < 0.001; ****p < 0.0001.

## AUTHOR CONTRIBUTIONS

**Yi Wang**: Conceptualization; methodology; software; investigation; validation; supervision; funding acquisition; writing—original draft; writing—review and editing; project administration. **Shuzi Liu**: Investigation; data curation; resources; formal analysis. **Laurence Don Wai Luu**: Formal analysis; visualization; writing—review and editing. **Yongzhi Zhai**: Investigation; resources; data curation; formal analysis. **Chenliang Zhu**: Investigation; visualization; project administration; formal analysis. **Zhaomin Feng**: Investigation; validation; formal analysis; visualization. **Yao Tan**: Investigation; validation. **Linglong Wan**: Formal analysis; data curation. **Jie Wang**: Formal analysis; data curation. **Juan Zhou**: Formal analysis; data curation. **Jing Wang**: Investigation; validation; resources; supervision. **Lixin Xie**: Investigation; validation; resources; project administration; supervision. **Quanyi Wang**: Conceptualization; visualization; project administration; supervision; writing—review and editing. **Fei Xie**: Conceptualization; supervision; investigation; validation; project administration; resources; writing—review and editing. All authors have read the final manuscript and approved it for publication.

## CONFLICT OF INTEREST STATEMENT

The authors declare no conflicts of interest.

## ETHICS STATEMENT

The ethical approval (No. 2022113030901836) for this study was obtained from the Ethics Committee of General Hospital of the People's Liberation Army of China.

## Supporting information


**Figure S1.** Quality control, demographic, and clinical data for the study cohort, related to Figure 1.
**Figure S2.** Identification and quantification of major immune cell lineages, related to Figure 1.
**Figure S3.** Identification and scoring of hyperinflammatory cell populations, related to Figure 2.
**Figure S4.** Expression patterns of key cytokines and associated signaling pathway components, related to Figure 2.
**Figure S5.** Detailed ligand‐receptor interactions among hyperinflammatory myeloid subsets in severe IAV infection, related to Figure 2.
**Figure S6.** Annotation and functional scoring of CD8^+^ T cell subsets, related to Figure 3.
**Figure S7.** Detailed transcriptional signatures of CD8^+^ T cells across different clinical states, related to Figure 3.
**Figure S8.** Characterization and functional scoring of CD4^+^ T cell subsets, related to Figure 4.
**Figure S9.** Transcriptional programs of pathogenic Treg and effector CD4^+^ T cell subsets, related to Figure 4.
**Figure S10.** Pathway analysis of effector CD4^+^ T cells, related to Figure 4.
**Figure S11.** Characterization of B cell subsets and their transcriptional programs, related to Figure 5.
**Figure S12.** Annotation and network analysis of myeloid subsets, related to Figure 6.
**Figure S13.** Hub cell identification and functional programs of classical monocytes, related to Figure 6.
**Figure S14.** Transcriptional programs of intermediate and non‐classical monocytes, related to Figure 6.
**Figure S15.** Pathogenic signatures of non‐classical monocytes and neutrophils, related to Figure 6.

**Table S1.** Detailed clinical information and laboratory findings of the enrolled individuals.
**Table S2.** Marker genes and signature genes for cell types, related to Figure 1 and Figure S3.
**Table S3.** Inflammatory genes and cytokine score, related to Figure 2.
**Table S4.** Specific upregulated DEGs from mild patients in CD8+T cells.
**Table S5.** Specific upregulated DEGs from severe patients in CD8+T cells.
**Table S6.** Specific upregulated DEGs from convalescent patients in CD8+T cells.
**Table S7.** Specific upregulated GO from mild patients in CD8+T cells.
**Table S8.** Specific upregulated GO from severe patients in CD8+T cells.
**Table S9.** Specific upregulated GO from convalescent patients in CD8+T cells.
**Table S10.** Specific upregulated DEGs from mild patients in CD4+ Te cells.
**Table S11.** Specific upregulated DEGs from severe patients in CD4+ Te cells.
**Table S12.** Specific upregulated DEGs from convalescent patients in CD4+ Te cells.
**Table S13.** Specific upregulated GO from mild patients in CD4+ Te cells.
**Table S14.** Specific upregulated GO from severe patients in CD4+ Te cells.
**Table S15.** Specific upregulated DEGs from mild patients in B (navie and memory) cells.
**Table S16.** Specific upregulated DEGs from severe patients in B (navie and memory) cells.
**Table S17.** Specific upregulated DEGs from convalescent patients in B (navie and memory) cells.
**Table S18.** Specific upregulated DEGs from mild patients in classical monocytes.
**Table S19.** Specific upregulated DEGs from severe patients in classical monocytes.
**Table S20.** Specific upregulated DEGs from convalescent patients in classical monocytes.

## Data Availability

The data that support the findings of this study are openly available in the China National Center for Bioinformation at https://ngdc.cncb.ac.cn/omix/release/OMIX014728 (Reference number OMIX014728). Supplementary materials (figures, tables, graphical abstract, slides, videos, Chinese translated version, and updated materials) may be found in the online DOI or iMeta Science http://www.imeta.science/.
