# Supplementary material for: A large‐scale single‐cell transcriptomic atlas indicates the immune panorama of influenza A infection
Source: Imeta. 2026 Mar 25;5(2):e70121. doi: 10.1002/imt2.70121 (PMC13147933; doi:10.1002/imt2.70121)
Supplement: Supplementary file 1 — Figure S1. Quality control, demographic, and clinical data for the study cohort, related to Figure 1. Figure S2. Identification and quantification of major immune cell lineages, related to Figure 1. Figure S3. Identification and scoring of hyperinflammatory cell populations, related to Figure 2. Figure S4. Expression patterns of key cytokines and associated signaling pathway components, related to Figure 2. Figure S5. Detailed ligand‐receptor interactions among hyperinflammatory myeloid subsets in severe IAV infection, related to Figure 2. Figure S6. Annotation and functional scoring of CD8+ T cell subsets, related to Figure 3. Figure S7. Detailed transcriptional signatures of CD8+ T cells across different clinical states, related to Figure 3. Figure S8. Characterization and functional scoring of CD4+ T cell subsets, related to Figure 4. Figure S9. Transcriptional programs of pathogenic Treg and effector CD4+ T cell subsets, related to Figure 4. Figure S10. Pathway analysis of effector CD4+ T cells, related to Figure 4. Figure S11. Characterization of B cell subsets and their transcriptional programs, related to Figure 5. Figure S12. Annotation and network analysis of myeloid subsets, related to Figure 6. Figure S13. Hub cell identification and functional programs of classical monocytes, related to Figure 6. Figure S14. Transcriptional programs of intermediate and non‐classical monocytes, related to Figure 6. Figure S15. Pathogenic signatures of non‐classical monocytes and neutrophils, related to Figure 6. [file IMT2-5-e70121-s002.docx]

**Supporting information to**

**A large-scale single-cell transcriptomic atlas indicates the immune panorama of influenza A infection**

**Running title:** Single-cell immune landscape of influenza

Yi Wang ^1,2#*^, Shuzi Liu ^3#^, Laurence Don Wai Luu ^4#^, Yongzhi Zhai ^5#^, Chenliang Zhu ^6#^, Zhaomin Feng ^7#^, Yao Tan ^8^, Linglong Wan ^1^, Jie Wang ^1^, Juan Zhou ^1^, Jing Wang ^9^, Lixin Xie ^8*^, Quanyi Wang ^7*^, Fei Xie ^8*^

^1^ Experimental Research Center & Molecular Diagnostic Center, Capital Center for Children's Health, Capital Medical University, Capital Institute of Pediatrics, Beijing 100020, China.

^2^ Beijing Research Center for Respiratory Infectious Diseases, Beijing 100020, China.

^3^ Department of Respiratory and Critical Care Medicine, The Eight Medical Center, Chinese PLA General Hospital, Beijing 100853, China

^4^ School of Biotechnology and Biomolecular Sciences, University of New South Wales, Sydney 2052, Australia

^5^ Department of Emergency, The First Medical Center, Chinese PLA General Hospital, Medical School of Chinese PLA, Beijing 100853, China.

^6^ Department of Clinical Laboratory, Institute of Translational Medicine, Renmin Hospital of Wuhan University, Wuhan 430060, China

^7^ Beijing Key Laboratory of Surveillance, Early Warning and Pathogen Research on Emerging Infectious Diseases, Beijing Research Center for Respiratory Infectious Diseases, Beijing Center for Disease Prevention and Control, Beijing 100013, China

^8^ College of Pulmonary and Critical Care Medicine, Chinese PLA General Hospital, Beijing 100091, China

^9^ Department of Respiratory and Critical Care Medicine, Beijing Chaoyang Hospital Affiliated to Capital Medical University, Beijing 100020, China

^#^ These authors contributed equally: Yi Wang, Kaifei Wang, Laurence Don Wai Luu, Yongzhi Zhai, Chenliang Zhu, Zhaomin Feng

^*^Correspondence: [wildwolf0101@163.com](mailto:wildwolf0101@163.com) (Yi Wang); [xielx301@126.com](mailto:xielx301@126.com) (Lixin Xie); [wangqy@bjcdc.org](mailto:wangqy@bjcdc.org) (Quanyi Wang); [xiefei0522@163.com](mailto:xiefei0522@163.com) (Fei Xie)

**
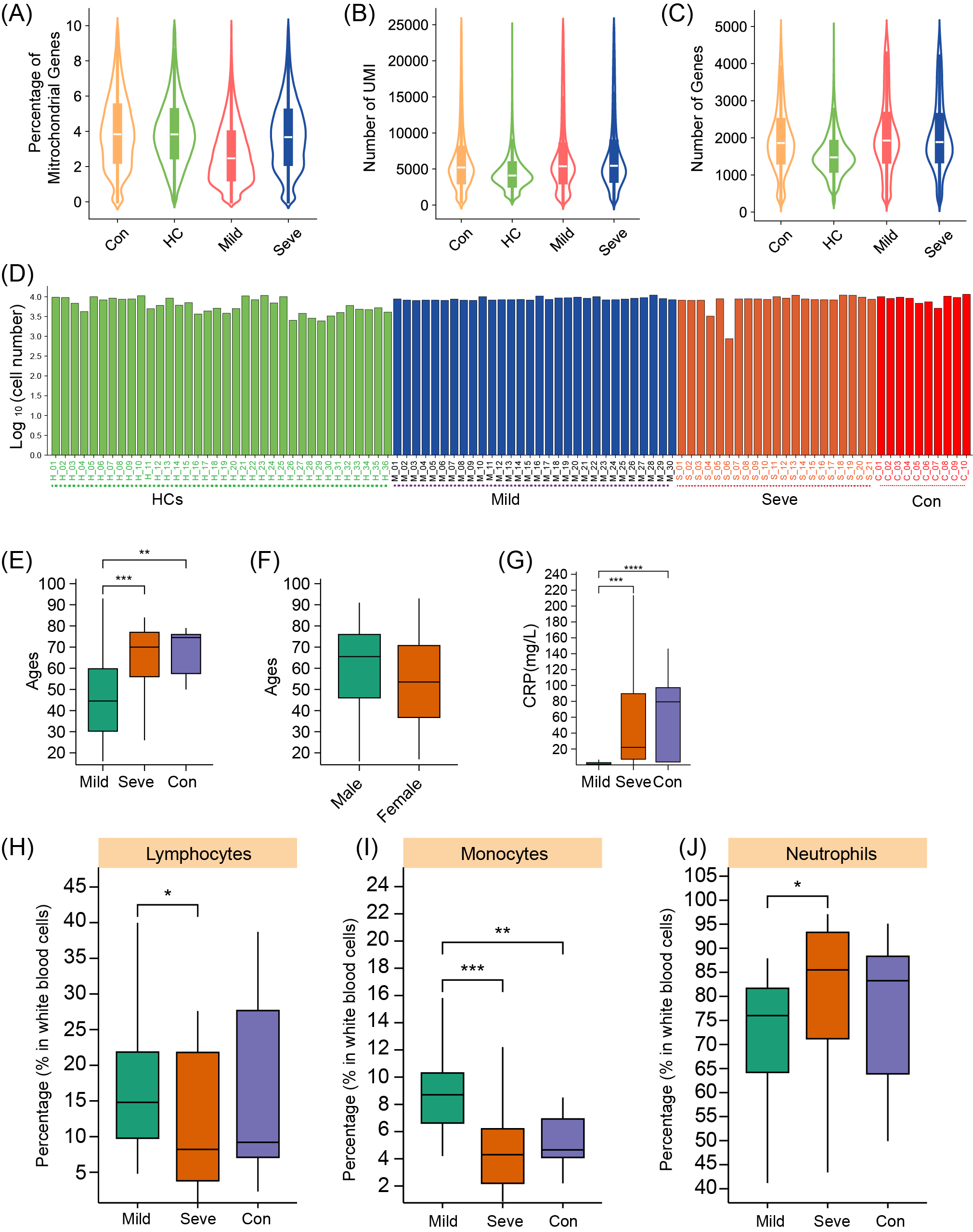
**

**Figure S1 Quality control, demographic, and clinical data for the study cohort, related to Figure 1.** (A-C) Violin plots showing key quality control metrics for single cells from each clinical group (Con, n = 10; HC, n = 36; Mild, n = 30; Seve, n = 21). Metrics include (A) percentage of mitochondrial genes, (B) number of unique molecular identifiers (UMIs), and (C) number of detected genes per cell. (D) Bar plot showing the logarithm (base 10) of the number of high-quality cells recovered from each of the 97 individual samples, grouped by clinical condition. Each bar represents one sample. (E) Box plot of patient age across the Mild, Severe, and Convalescent groups. (F) Box plot of donor ages across male and female individuals in the entire cohort. (G) Box plot of C-reactive protein (CRP) levels (mg/L) in patients from the Mild, Severe, and Convalescent groups. (H-J) Box plots showing the percentage of major leukocyte populations from clinical complete blood counts for the indicated patient groups. Proportions are shown for (H) Lymphocytes, (I) Monocytes, and (J) Neutrophils as a percentage of total white blood cells. For all box plots, the center line represents the median, the box limits represent the upper and lower quartiles, and the whiskers extend to 1.5 times the interquartile range. Asterisks denote statistical significance (**p* < 0.05, ***p* < 0.01, ****p* < 0.001, *****p* < 0.0001) as determined by Kruskal-Wallis test with Bonferroni correction.

**
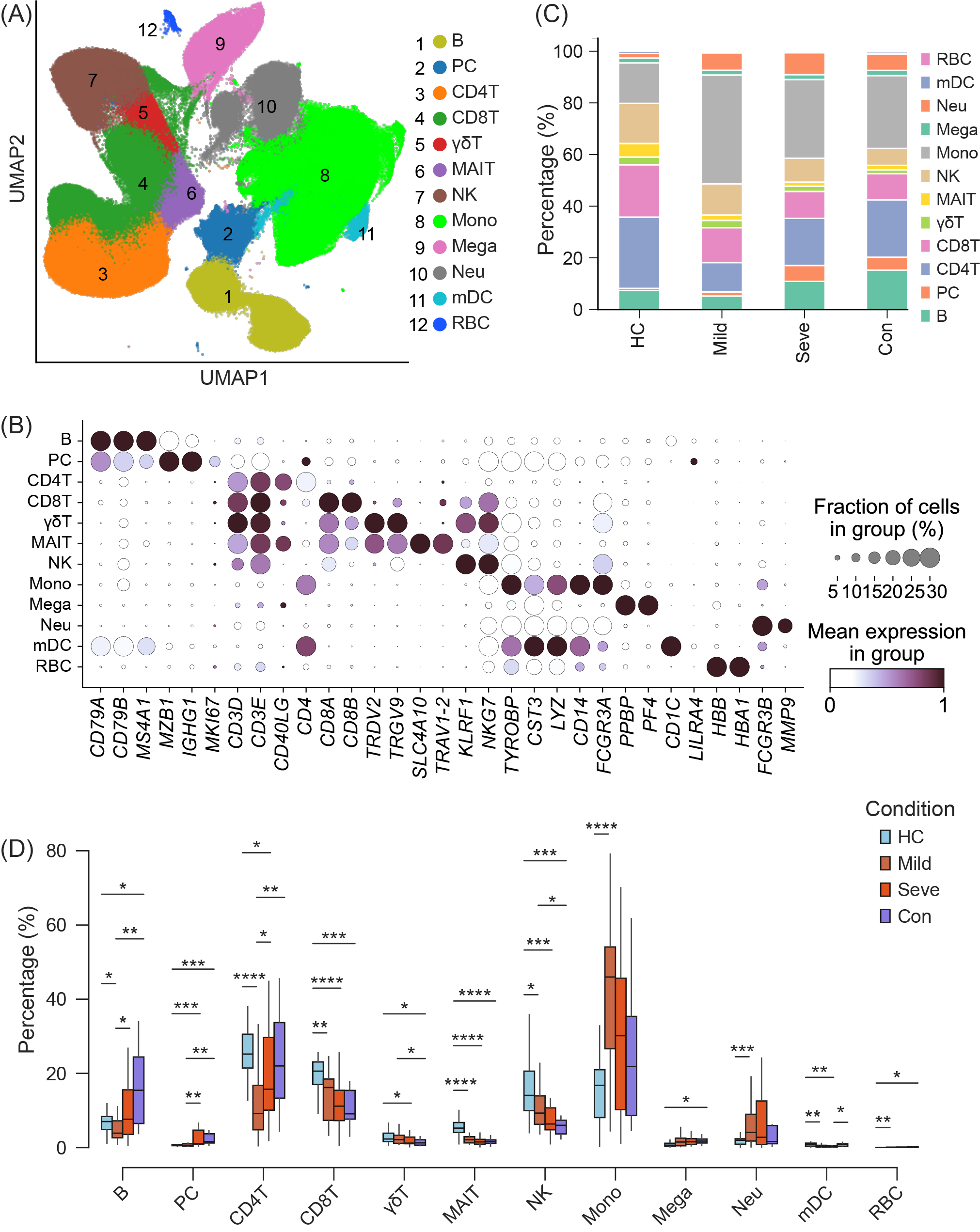
**

**Figure S2 Identification and quantification of major immune cell lineages, related to Figure 1.** (A) UMAP projection of all 612,010 cells, with colors corresponding to 12 major immune cell lineages identified through unsupervised clustering. B, B cells; PC, plasma cells; CD4T, CD4^+^ T cells; CD8T, CD8^+^ T cells; γδT, gamma delta T cells; MAIT, Mucosal-associated invariant T cells; NK, natural killer cells; Mono, monocytes; Mega, megakaryocytes; Neu, neutrophils; mDC, myeloid dendritic cells; RBC, red blood cells. (B) Dot plot displaying the expression of canonical marker genes used to annotate the 12 major cell lineages shown in (A). The dot size indicates the percentage of cells within a lineage expressing the gene, while the color intensity represents the average expression level. (C) Stacked bar plot illustrating the relative proportions of the 12 major cell lineages within each of the four clinical groups (HC, Mild, Seve, Con). (D) Box plots quantifying the percentage of each major cell lineage across the four clinical conditions. The center line represents the median, box limits represent upper and lower quartiles, and whiskers extend to 1.5 times the interquartile range. Statistical significance was determined using Kruskal-Wallis test with Bonferroni correction (**p* < 0.05, ***p* < 0.01, ****p* < 0.001, *****p* < 0.0001).

**
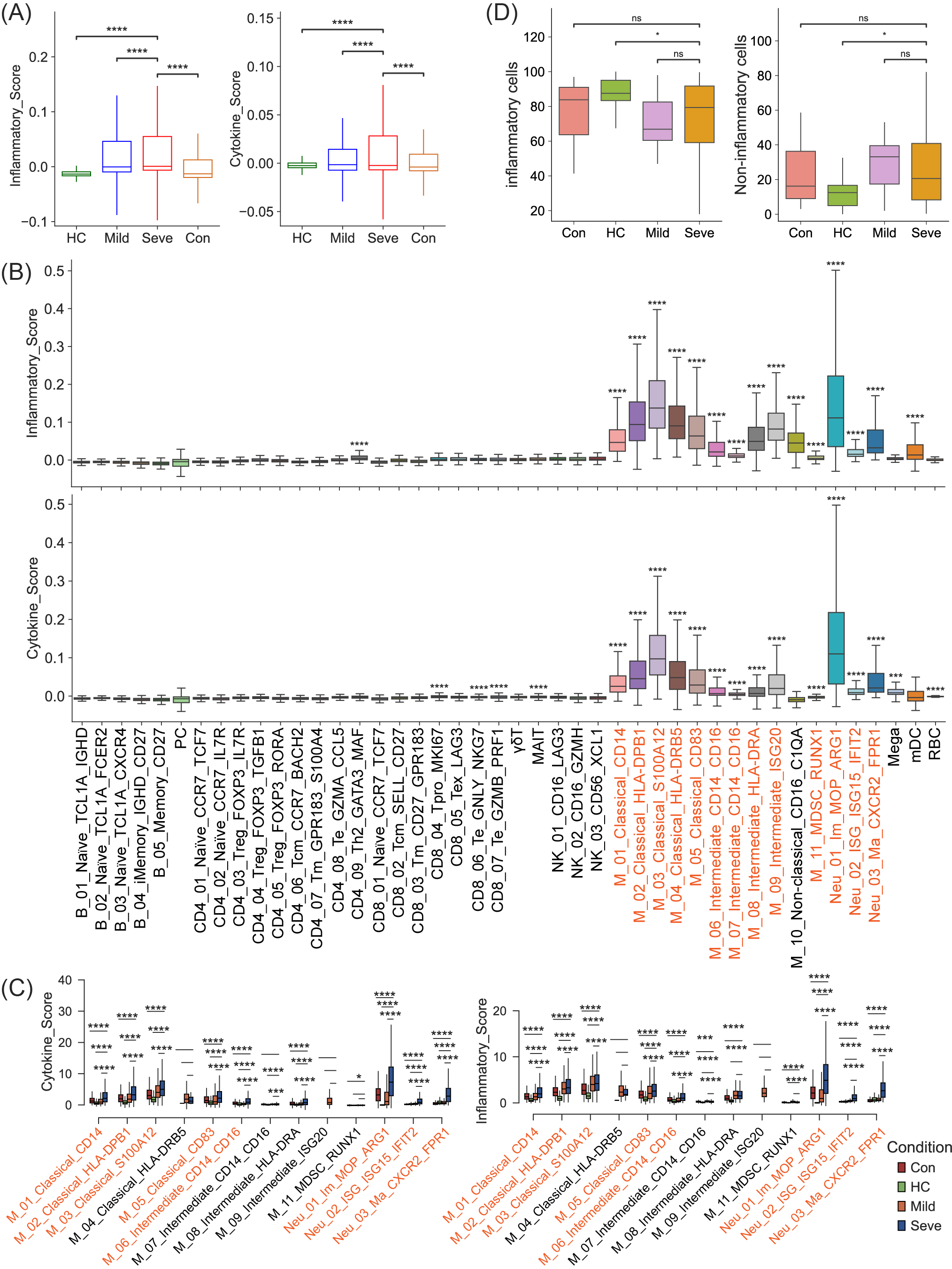
**

**Figure S3 Identification and scoring of hyperinflammatory cell populations, related to Figure 2.** (A) Box plots comparing the overall Inflammatory Score (left) and Cytokine Score (right) calculated across all PBMCs for each of the four clinical groups (HC, Mild, Seve, Con). (B) Box plots showing the distribution of the Inflammatory Score (top) and Cytokine Score (bottom) for all 44 identified cell subsets. Subsets with significantly elevated scores, predominantly myeloid lineages, are highlighted with red text. (C) Box plots comparing the Cytokine Score (top) and Inflammatory Score (bottom) for the eight selected hyperinflammatory myeloid subsets across the four clinical conditions. (D) Box plots comparing the absolute counts of inflammatory (left) and non-inflammatory (right) cells per sample across the four clinical groups. For all box plots, the center line represents the median, the box limits represent the upper and lower quartiles, and the whiskers extend to 1.5 times the interquartile range. Asterisks denote statistical significance (**p* < 0.05, ***p* < 0.01, ****p* < 0.001, *****p* < 0.0001; ns, not significant) as determined by Kruskal-Wallis test with Bonferroni correction.

**
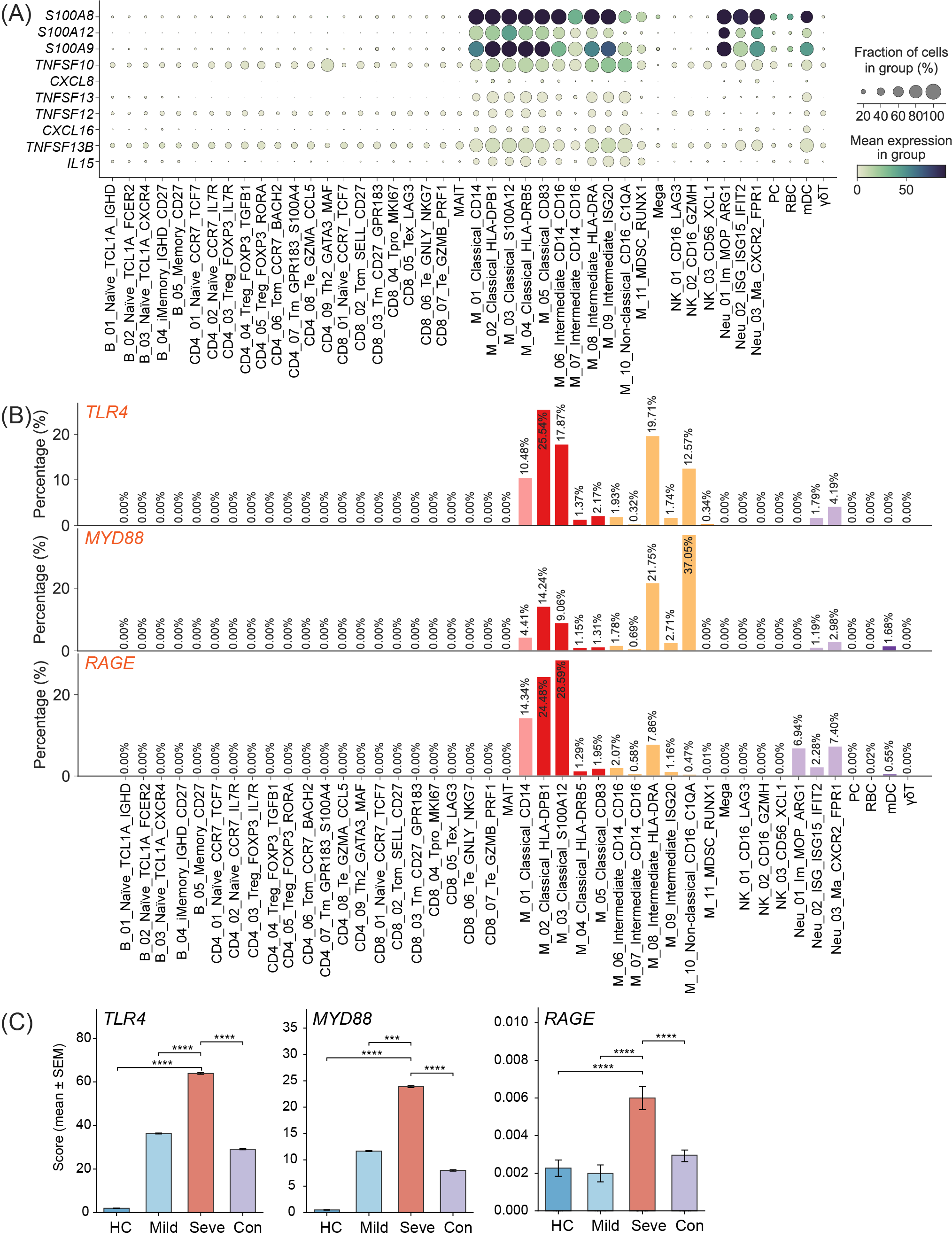
**

**Figure S4 Expression patterns of key cytokines and associated signaling pathway components, related to Figure 2.** (A) Dot plot showing the expression of the ten cytokines that contribute most significantly to the total cytokine score across all 44 cell subsets. Dot size indicates the percentage of cells expressing the gene, while color intensity represents the average expression level. (B) Bar plots displaying the percentage of cells expressing key genes of the *S100*-*TLR4*/*RAGE* signaling pathway: *TLR4* (top), *MYD88* (middle), and *AGER* (encoding *RAGE*, bottom) across all 44 cell subsets. Expression is predominantly localized to monocyte and neutrophil populations. (C) Bar plots displaying the statistical comparison of expression scores for the key signaling receptors *TLR4*, *MYD88*, and *RAGE* (*AGER*) across the four clinical groups (HC, Mild, Severe, Con). Data are presented as mean ± SEM. The expression of all three components is significantly elevated in the Severe group. Statistical significance was determined using the Kruskal-Wallis test with Bonferroni correction (*****p* < 0.0001, ****p* < 0.001).

**
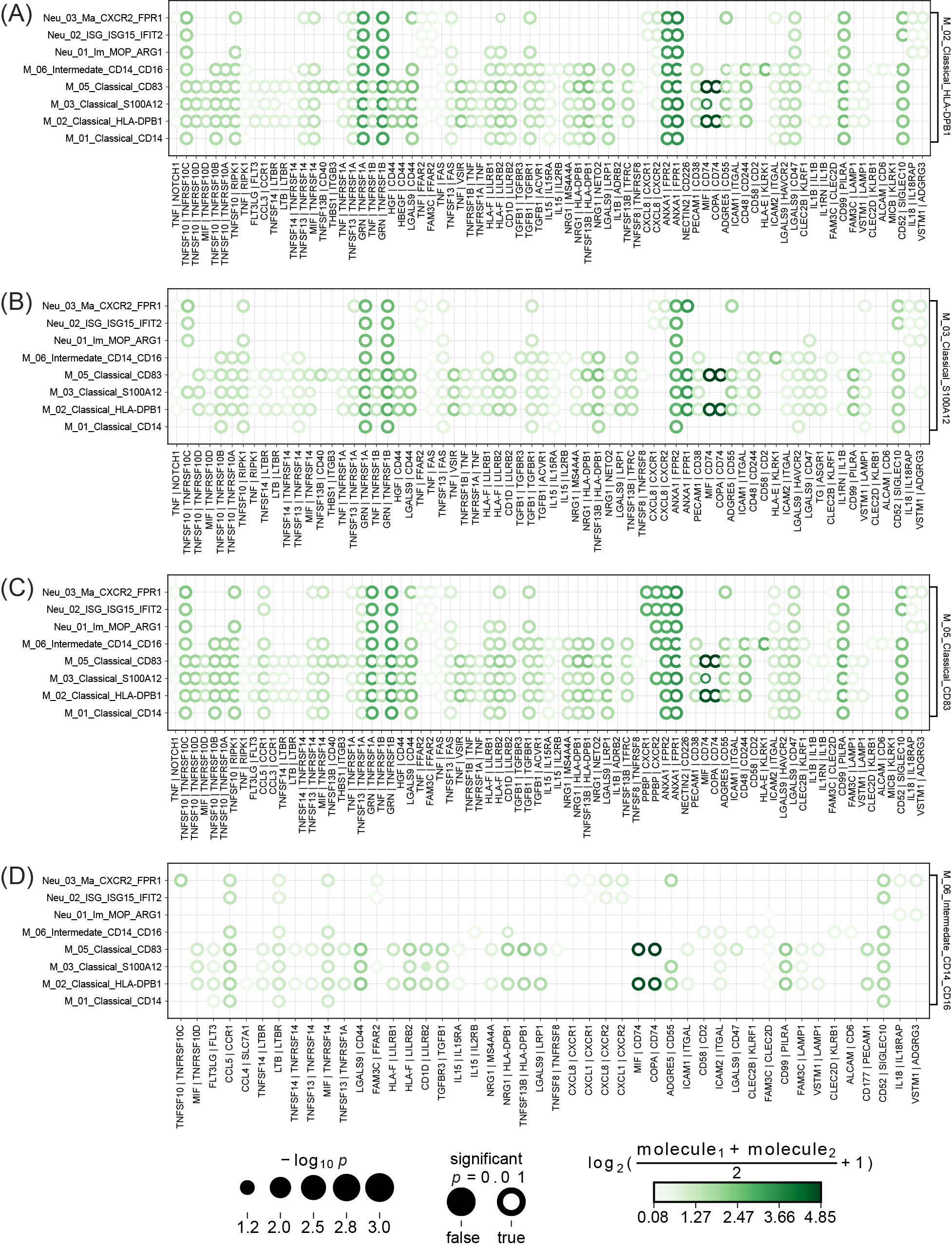
**

**Figure S5 Detailed ligand-receptor interactions among hyperinflammatory myeloid subsets in severe IAV infection, related to Figure 2.** (A-D) Dot plots detailing significant ligand-receptor (L-R) interactions among the eight hyperinflammatory myeloid subsets in severe patients. The plots show interactions where the sender cell populations are listed on the y-axis, and the receiver populations are indicated in the clipped titles at the top right of each panel. The L-R pairs are shown on the x-axis. The color of the dots represents the log2-transformed mean expression of the interacting molecule pair, and the size of the dots corresponds to the statistical significance (-log10 *p*-value). *p*-values were calculated using a permutation test. These panels collectively illustrate the dense communication network driving the inflammatory state.

**
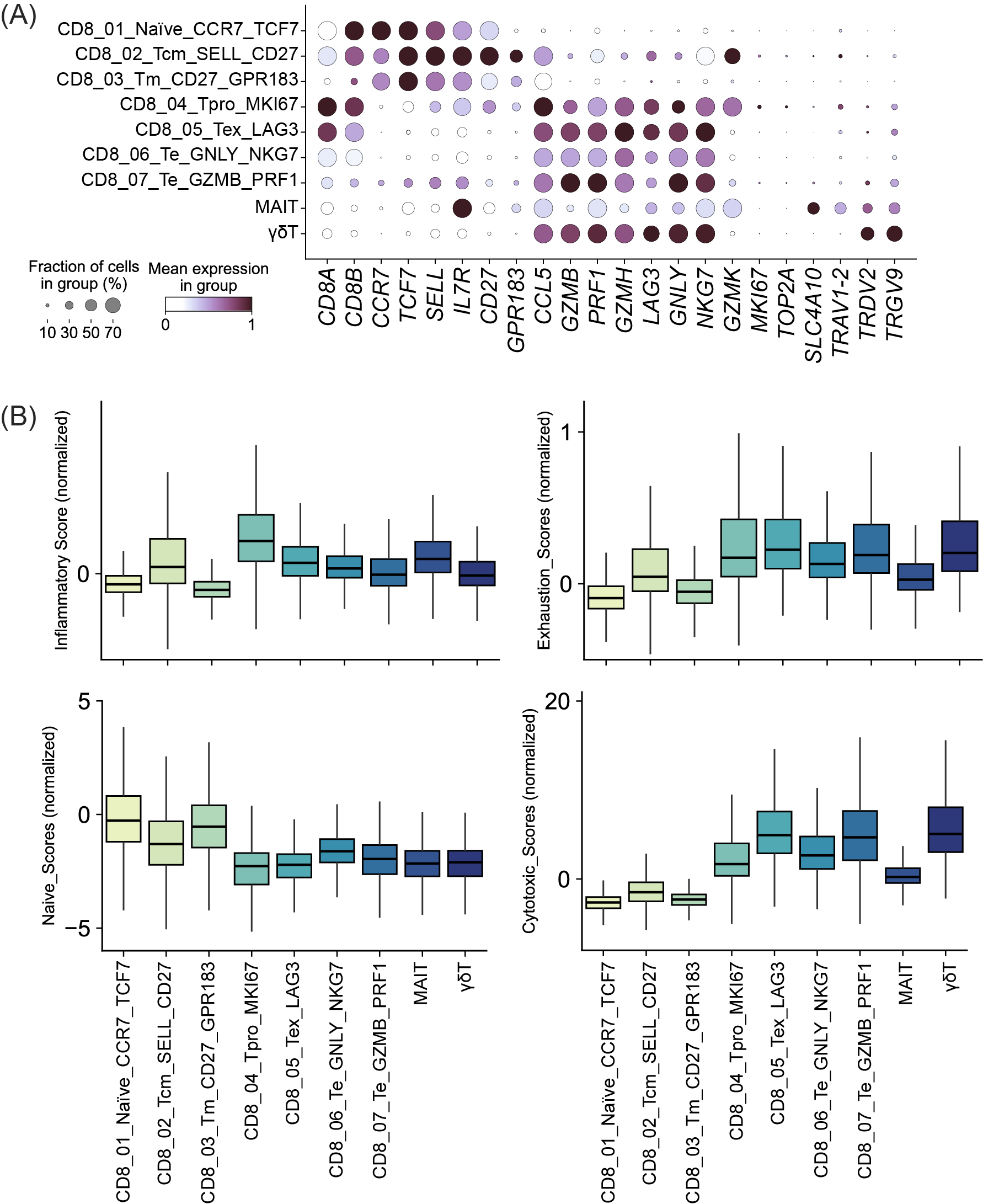
**

**Figure S6 Annotation and functional scoring of CD8^+^ T cell subsets, related to Figure 3.** (A) Dot plot showing the expression of canonical marker genes used to annotate the nine CD8^+^ T cell, MAIT, and γδ T cell subsets. The dot size corresponds to the percentage of cells within a subset expressing the gene, while the color intensity represents the average expression level. (B) Box plots displaying normalized module scores for four distinct functional states across the nine identified subsets: Inflammatory Score (top left), Exhaustion Score (top right), Naïve Score (bottom left), and Cytotoxic Score (bottom right). The center line represents the median, the box limits represent the upper and lower quartiles, and the whiskers extend to 1.5 times the interquartile range.

**
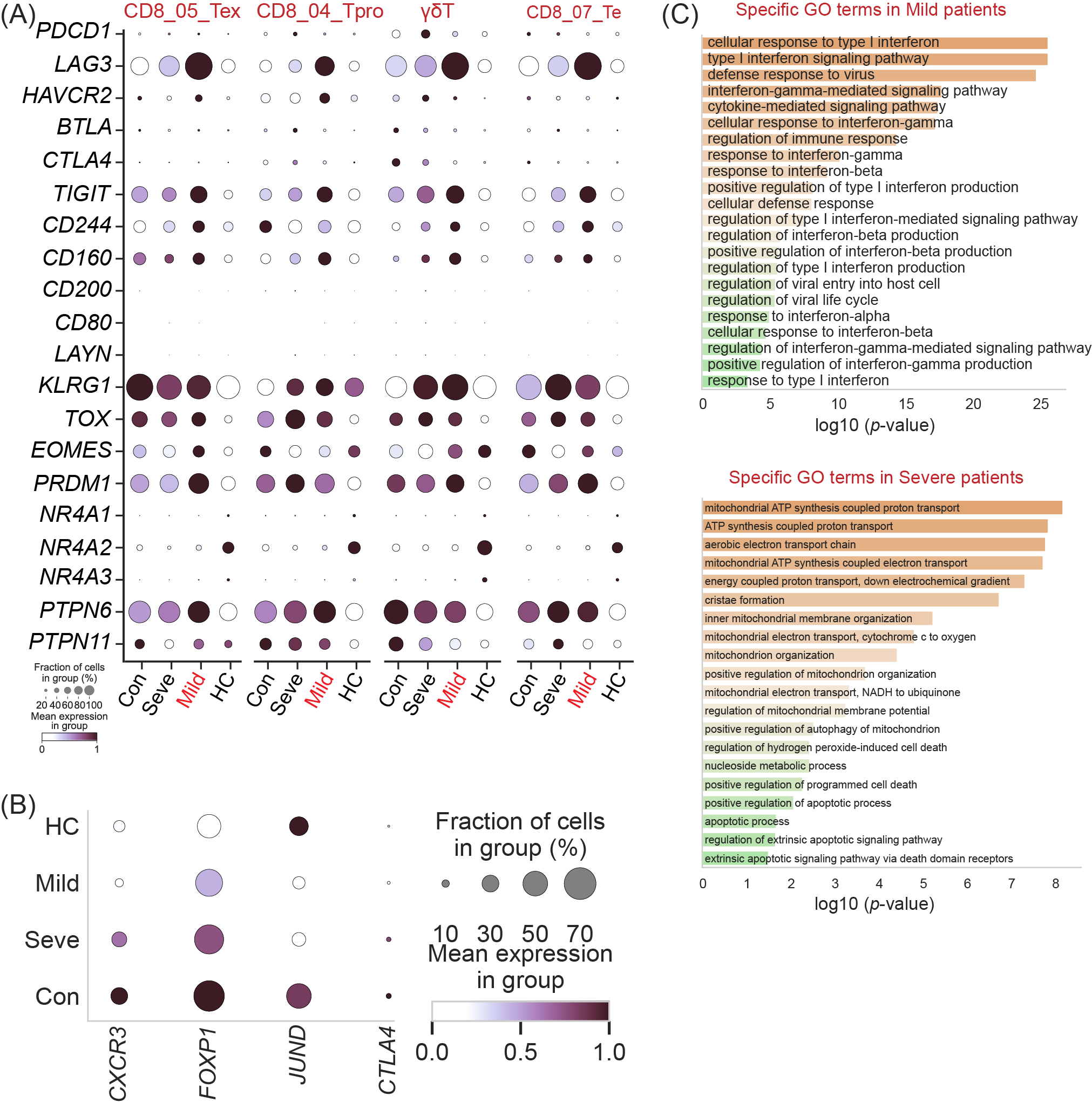
**

**Figure S7 Detailed transcriptional signatures of CD8^+^ T cells across different clinical states, related to Figure 3.** (A) Dot plot showing the expression of canonical exhaustion markers (top) and associated transcription factors (bottom) in four key CD8^+^ T cell and γδ T cell subsets across the four clinical groups. Dot size indicates the percentage of cells expressing the gene, and color intensity reflects the average expression level. (B) Dot plot showing the expression of genes associated with T cell memory and immune quiescence (e.g., *CXCR3*, *FOXP1*, *CTLA4*) across the four clinical groups. (C) Bar plots displaying Gene Ontology (GO) enrichment analysis of upregulated DEGs in CD8^+^ T cells from Mild patients (top) and Severe patients (bottom). The x-axis represents the -log10(*p*-value) of the enrichment. Key pathways related to antiviral response (Mild) and mitochondrial dysfunction/apoptosis (Severe) are highlighted.

**
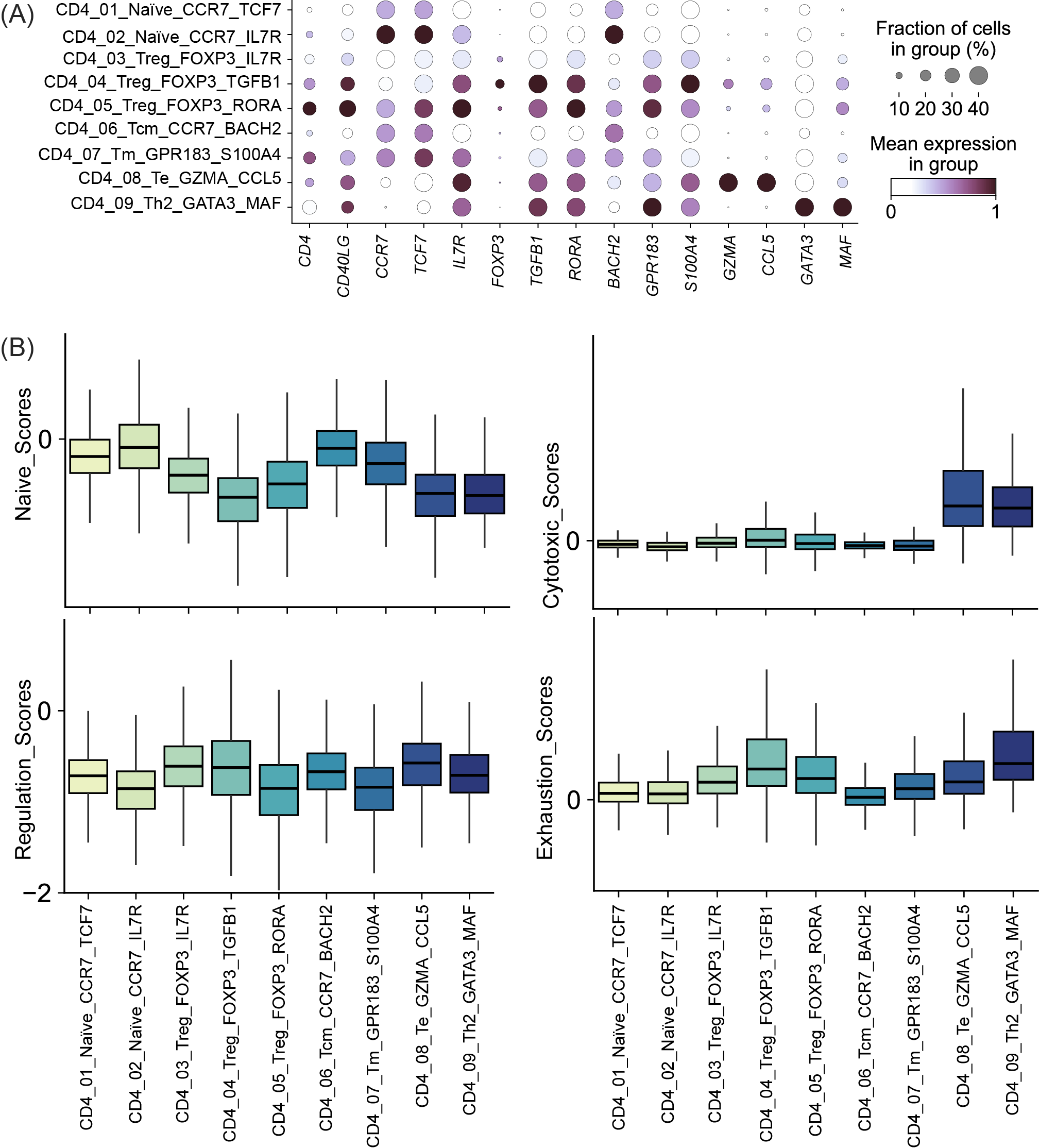
**

**Figure S8 Characterization and functional scoring of CD4^+^ T cell subsets, related to Figure 4.** (A) Dot plot showing the expression of canonical marker genes used to annotate the nine CD4^+^ T cell subsets. The dot size corresponds to the percentage of cells within a subset expressing the gene, while the color intensity represents the average expression level. (B) Box plots displaying normalized module scores for four distinct functional states across the nine identified subsets: Naïve Score (top left), Cytotoxic Score (top right), Regulation Score (bottom left), and Exhaustion Score (bottom right). The center line represents the median, the box limits represent the upper and lower quartiles, and the whiskers extend to 1.5 times the interquartile range.

**
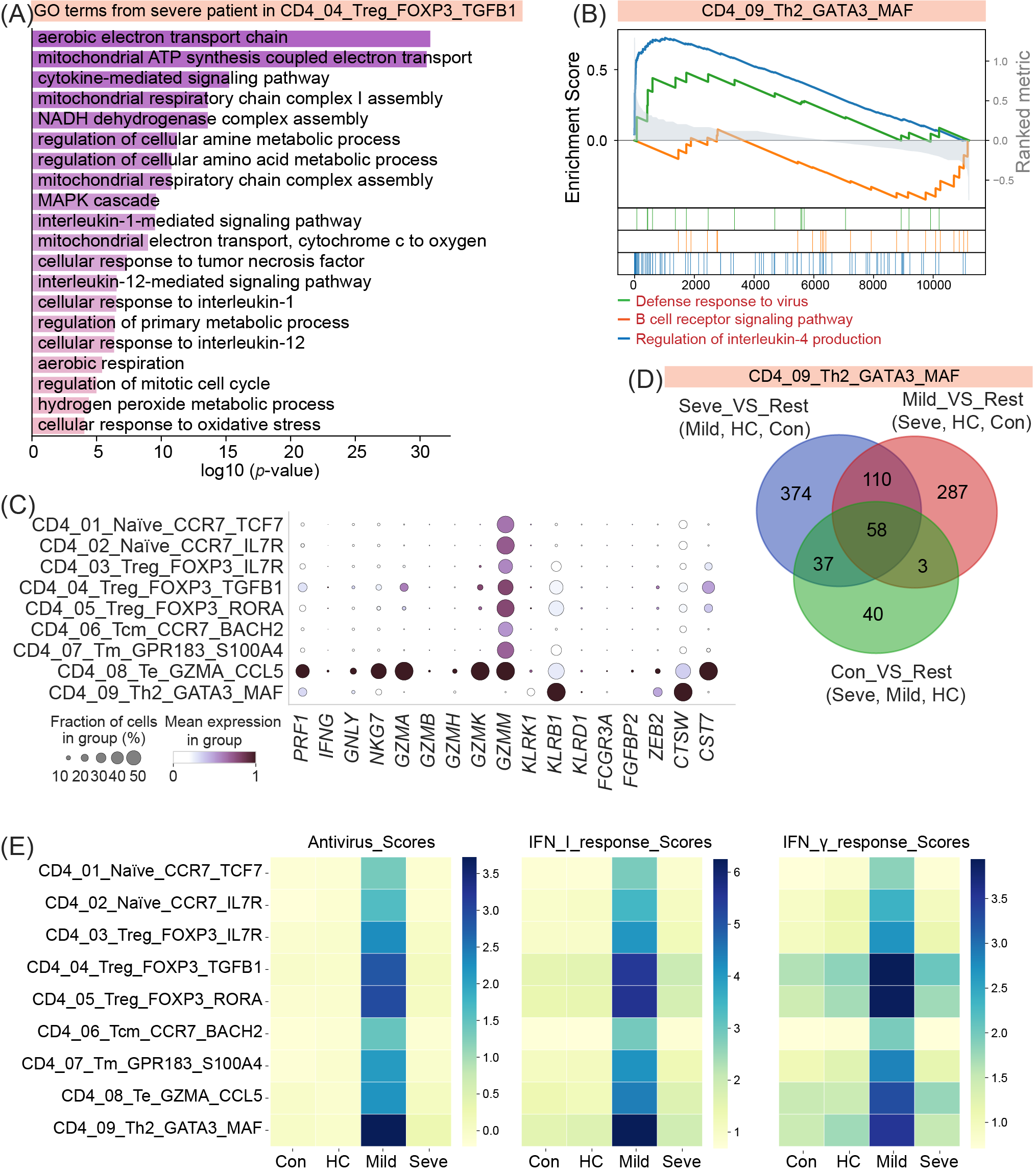
**

**Figure S9 Transcriptional programs of pathogenic Treg and effector CD4^+^ T cell subsets, related to Figure 4.** (A) Bar plot of Gene Ontology (GO) terms enriched in upregulated DEGs from the CD4_04_Treg_FOXP3_TGFB1 subset in severe patients. The x-axis represents the -log10(P-value). (B) GSEA plot showing enrichment of pathways related to viral defense and B cell signaling in CD4_09_Th2_GATA3_MAF cells from mild patients compared to the rest. (C) Dot plot showing the expression of key cytotoxic genes across all nine CD4^+^ T cell subsets. Dot size indicates the percentage of cells expressing the gene, and color intensity reflects the average expression level. (D) Venn diagram illustrating the overlap of upregulated DEGs in the CD4_09_Th2_GATA3_MAF subset from Mild, Severe, and Convalescent patients compared to the remaining groups (Rest). (E) Heatmaps showing the average module scores for Antivirus, Type I Interferon (IFN-I) response, and IFN-γ response across all nine CD4^+^ T cell subsets in each clinical group.

**
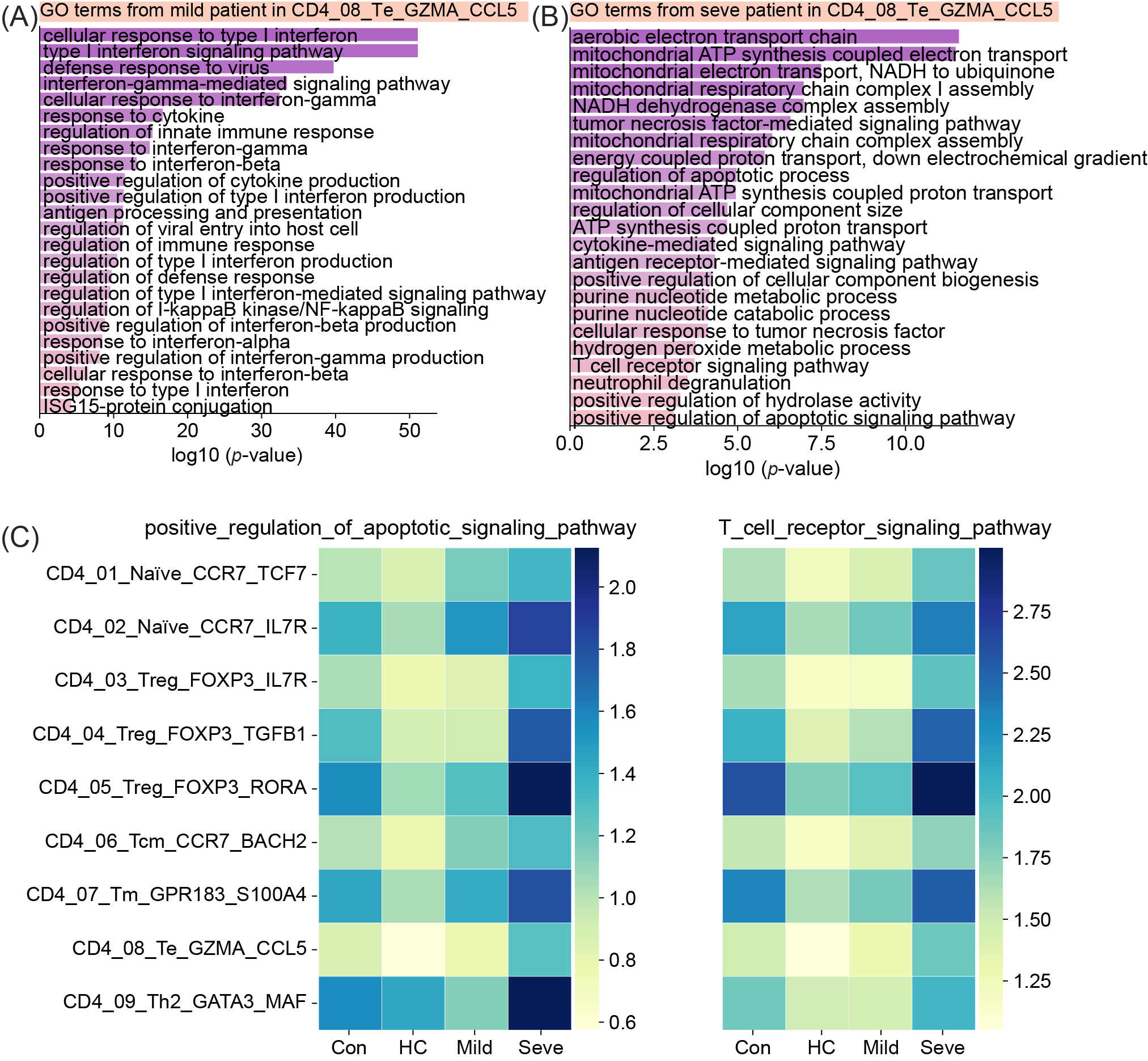
**

**Figure S10 Pathway analysis of effector CD4^+^ T cells, related to Figure 4.** (A and B) Bar plots showing enriched Gene Ontology (GO) terms for upregulated DEGs in the CD4_08_Te_GZMA_CCL5 subset from (A) Mild patients and (B) Severe patients. The x-axis represents the -log10(P-value). (C) Heatmaps displaying the average module scores for the 'positive regulation of apoptotic signaling pathway' (left) and 'T cell receptor signaling pathway' (right) across all nine CD4^+^ T cell subsets in the four clinical groups. The color scale indicates the score intensity.


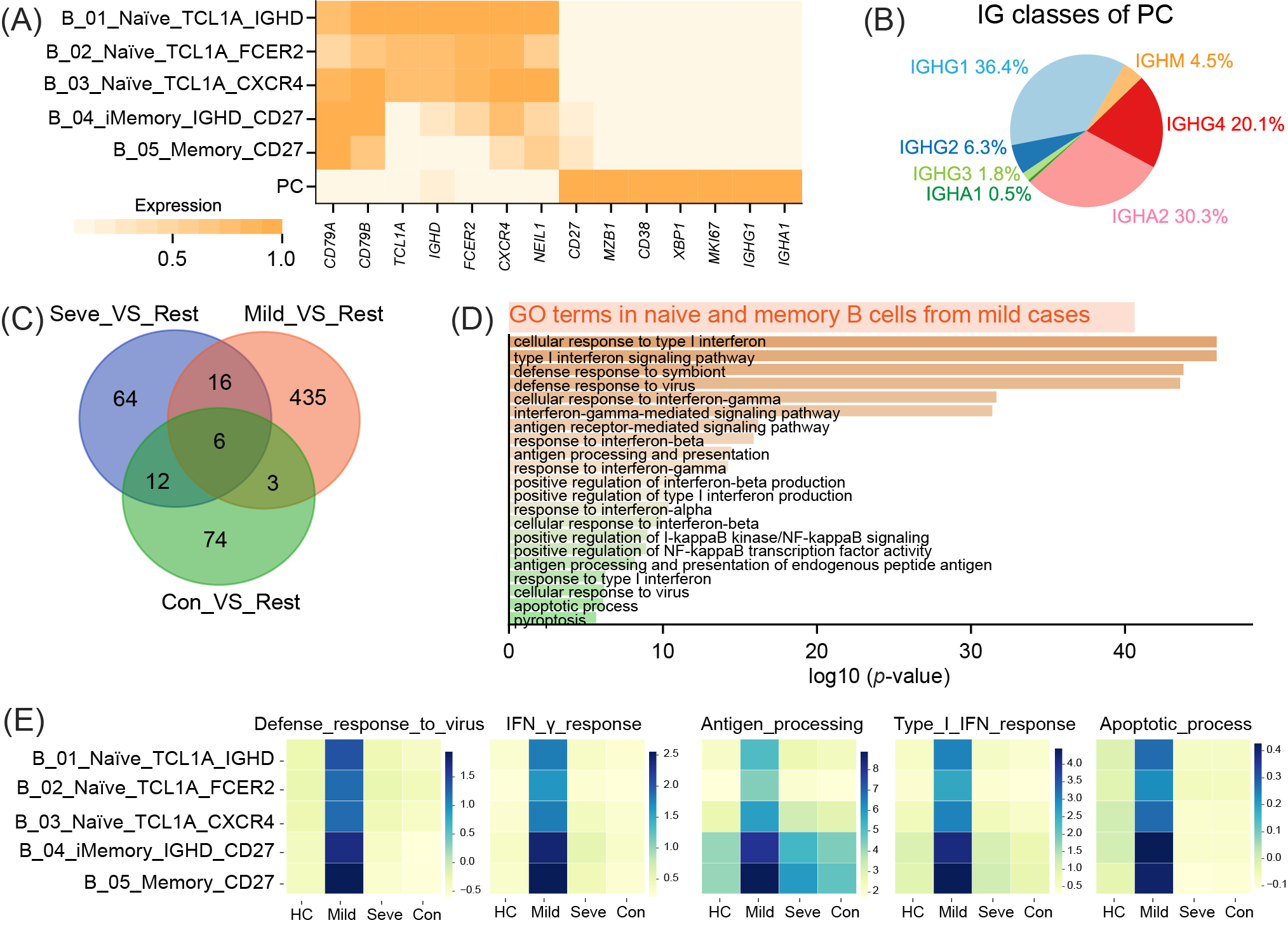


**Figure S11 Characterization of B cell subsets and their transcriptional programs, related to Figure 5.** (A) Heatmap displaying the expression of canonical marker genes used to annotate the six B cell and PC subsets shown in Fig 5A. (B) Pie chart illustrating the relative proportions of immunoglobulin (IG) classes expressed by plasma cells, based on transcript abundance. (C) Venn diagram showing the overlap of uniquely upregulated DEGs in naïve and memory B cells (subsets B_01-B_05) from Severe, Mild, and Convalescent patients compared to the remaining groups (Rest). (D) Bar plot of enriched Gene Ontology (GO) terms for upregulated DEGs in naïve and memory B cells from mild patients, highlighting pathways related to antiviral defense and immune signaling. (E) Heatmaps showing the average module scores for key biological pathways (Defense response to virus, IFN-γ response, Antigen processing, Type I IFN response, and Apoptotic process) across the five naïve and memory B cell subsets in each clinical group. The color scale indicates the score intensity.


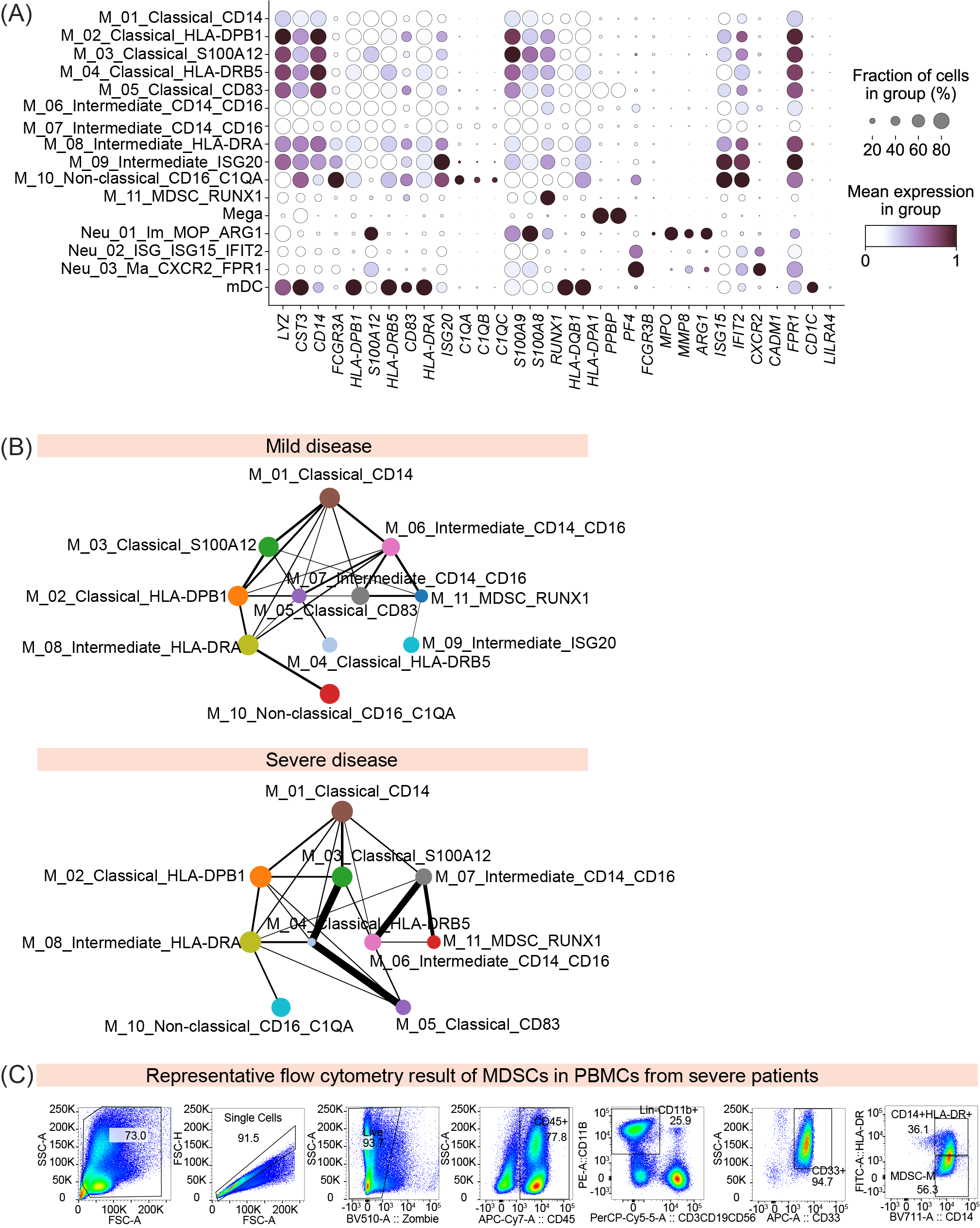


**Figure S12 Annotation and network analysis of myeloid subsets, related to Figure 6.** (A) Dot plot showing the expression of canonical marker genes used to annotate the 16 myeloid cell subsets. The dot size corresponds to the percentage of cells expressing the gene, while the color intensity represents the average expression level. (B) PAGA graphs illustrating the inferred connectivity network among monocyte subsets in mild disease (top) and severe disease (bottom). Line thickness indicates the strength of the connection, revealing a highly interconnected network in mild disease that is rewired and restricted in severe disease. (C) Representative flow cytometry plots validating the presence of Monocytic Myeloid-Derived Suppressor Cells (mMDSCs) in PBMCs from patients with severe IAV infection. The gating strategy identifies mMDSCs as Live, CD45+, Lineage (CD3/CD19/CD56/CD66b)-negative, CD14-positive, and HLA-DR-low/negative cells (CD14+HLA-DR-/low). The numbers in the plots indicate the percentage of cells within the parent gate. This protein-level validation confirms the expansion of the phenotypic mMDSC population corresponding to the M_11_MDSC_RUNX1 scRNA-seq cluster.


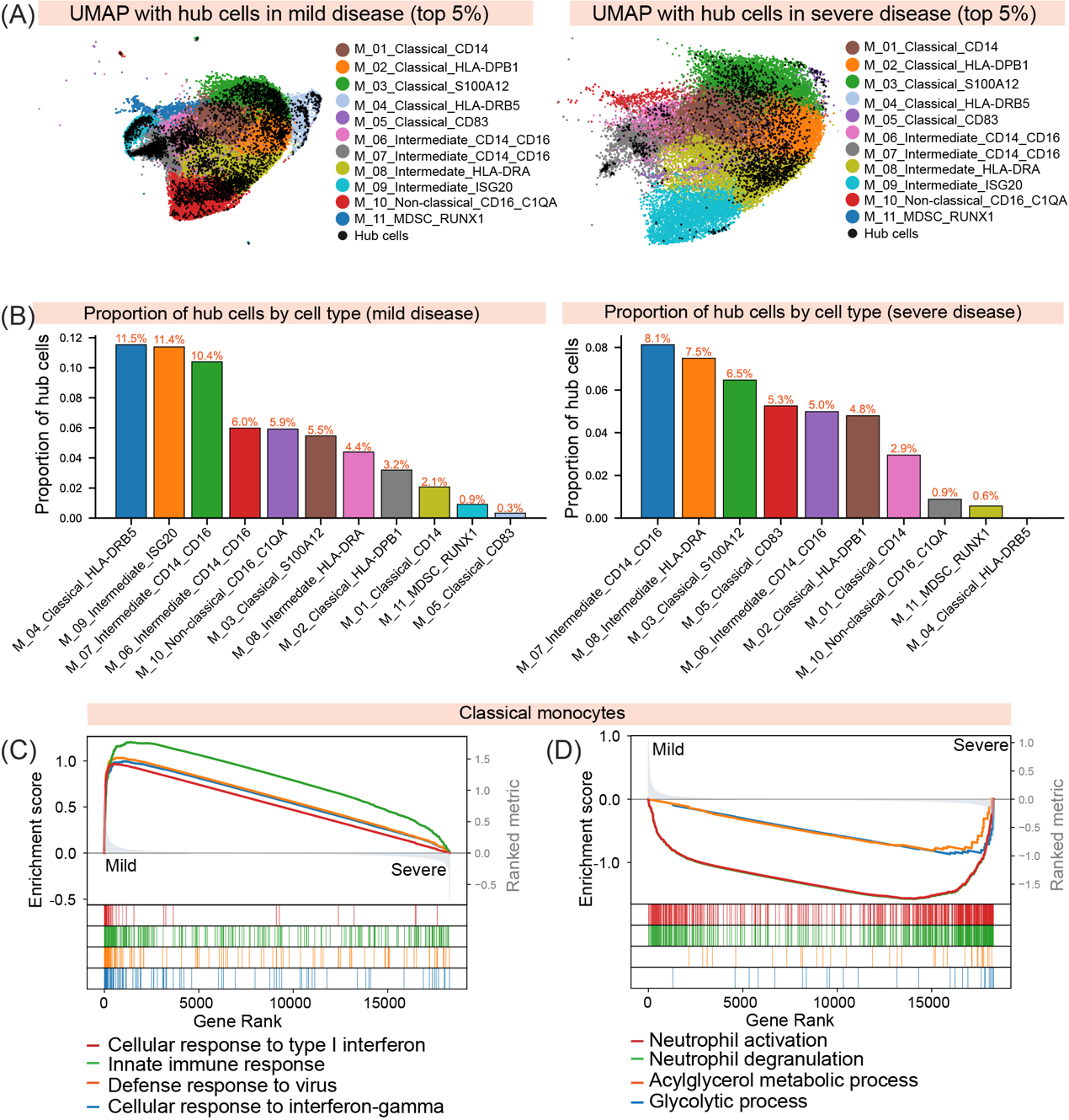


**Figure S13 Hub cell identification and functional programs of classical monocytes, related to Figure 6.** (A) UMAP plots highlighting the top 5% of monocytes with the highest PAGA connectivity (hub cells, colored blue) in mild disease (left) and severe disease (right). (B) Bar plots showing the proportional contribution of each monocyte subset to the hub cell population in mild (left) and severe (right) disease. (C and D) GSEA plots for classical monocytes (M_01-M_05) comparing mild versus severe patients, showing enrichment of (C) antiviral/immune response pathways in mild disease and (D) neutrophil activation/metabolic pathways in severe disease.

**
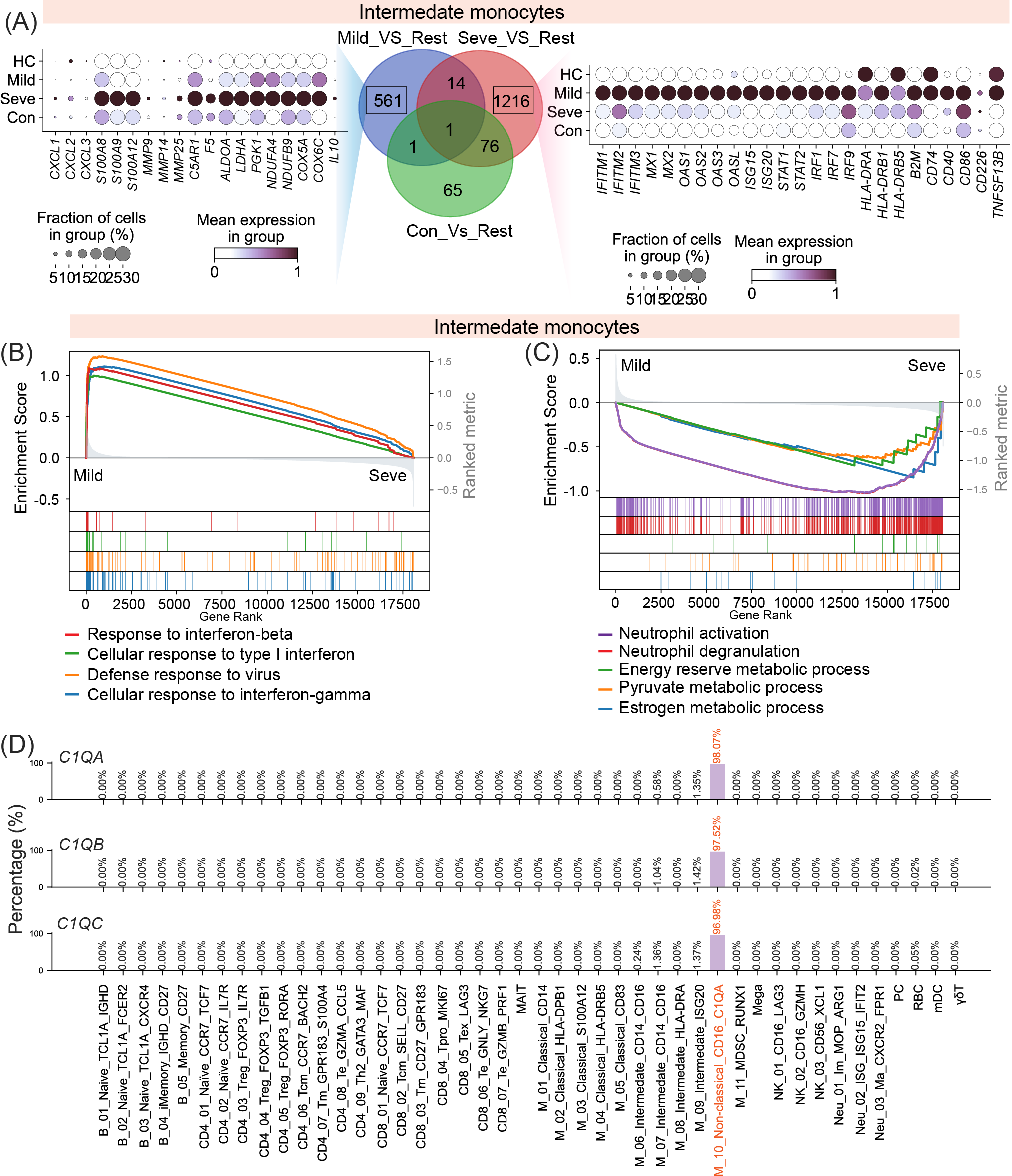
**

**Figure S14 Transcriptional programs of intermediate and non-classical monocytes, related to Figure 6.** (A) Left: Venn diagram of upregulated DEGs in intermediate monocytes (M_06-M_09) from Severe, Mild, and Convalescent patients. Right panels: Dot plots displaying representative genes from the Severe-specific pathogenic signature (top) and the Mild-specific protective signature (bottom). (B and C) GSEA plots for intermediate monocytes comparing mild versus severe patients, showing enrichment of (B) interferon/antiviral pathways in mild disease and (C) neutrophil activation/metabolic pathways in severe disease. (D) Bar plots showing the percentage of cells expressing the three core C1 complement component genes (*C1QA*, *C1QB*, *C1QC*) across all 44 cell subsets, identifying non-classical monocytes as the primary source.


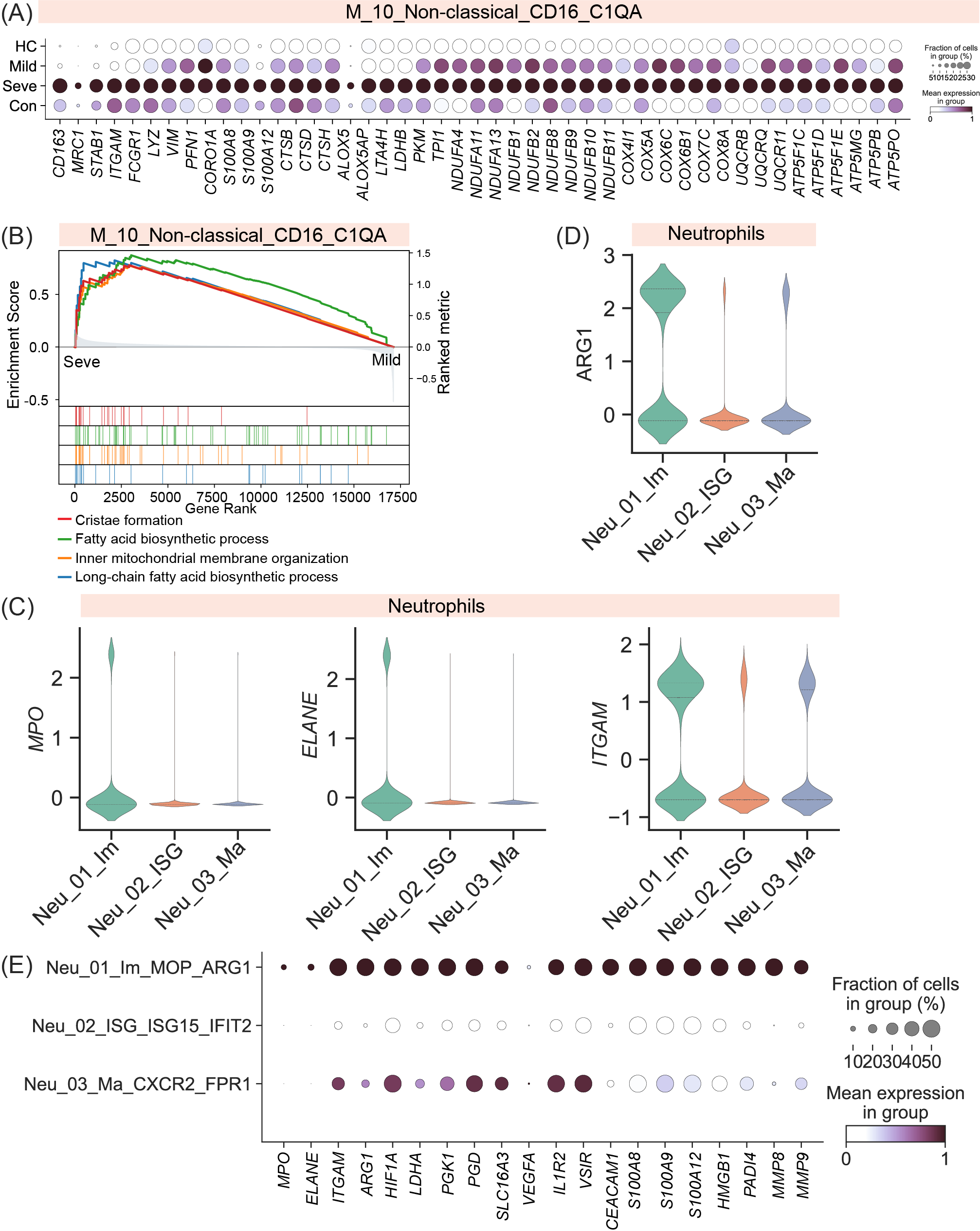


**Figure S15 Pathogenic signatures of non-classical monocytes and neutrophils, related to Figure 6.** (A) Dot plot displaying the expression of representative genes associated with the pathogenic signature (scavenger receptor signaling, phagocytosis, inflammation, and metabolic stress) of non-classical monocytes (M_10) across the four clinical groups. (B) GSEA plot for non-classical monocytes comparing severe versus mild patients, showing positive enrichment of pathways related to mitochondrial organization and metabolism. (C) Violin plots showing the expression of canonical neutrophil immaturity markers (*MPO*, *ELANE*) and the integrin ITGAM across the three identified neutrophil subsets. (D) Violin plot showing high expression of the immunosuppressive enzyme *ARG1* in the immature neutrophil subset (Neu_01_Im). (E) Dot plot displaying the expression of key genes associated with the immunosuppressive program of the immature neutrophil subset (Neu_01_Im_MOP_ARG1), including metabolic regulators, inhibitory molecules, and inflammatory mediators.
